# Pyruvate kinase isoform M2 impairs cognition in systemic lupus erythematosus by promoting microglial synaptic pruning via the β-catenin signaling pathway

**DOI:** 10.1186/s12974-021-02279-9

**Published:** 2021-10-13

**Authors:** Li Lu, Hailin Wang, Xuan Liu, Liping Tan, Xiaoyue Qiao, Jiali Ni, Yang Sun, Jun Liang, Yayi Hou, Huan Dou

**Affiliations:** 1 The State Key Laboratory of Pharmaceutical Biotechnology, Division of Immunology, Medical School, Nanjing University, Nanjing, 210093 People’s Republic of China; 2 Jiangsu Key Laboratory of Molecular Medicine, Nanjing, 210093 People’s Republic of China; 3 Department of Rheumatology and Immunology, Nanjing Drum Tower Hospital, The Affiliated Hospital of Nanjing University Medical School, Nanjing, 210008 People’s Republic of China; 4 The State Key Laboratory of Pharmaceutical Biotechnology and Collaborative Innovation Center of Chemistry for Life Sciences, School of Life Sciences, Nanjing University, Nanjing, 210023 China

**Keywords:** Systemic lupus erythematosus encephalopathy, Aerobic glycolysis, Pyruvate kinase isoform M2, Microglia, Phagocytosis, LC–MS analysis

## Abstract

**Background:**

Neuropsychiatric systemic lupus erythematosus (NPSLE) is a severe complication, which involves pathological damage to the brain and cognitive function. However, its exact mechanism of action still remains unclear. In this study, we explored the role of microglia in the cognitive dysfunction of NPSLE mice. We also analyzed and compared the metabolites in the hippocampal tissues of the lupus model and control mice.

**Methods:**

MRL/MpJ-Fas^lpr^ (MRL/lpr) female mice were used as the NPSLE mouse model. Metabolomics was used to assess hippocampal glycolysis levels. Glucose, lactic acid, IL-6, and IL-1β of the hippocampus were detected by ELISA. Based on the glycolysis pathway, we found that pyruvate kinase isoform M2 (PKM2) in the hippocampus was significantly increased. Thus, the expression of PKM2 was detected by qRT-PCR and Western blotting, and the localization of PKM2 in microglia (IBA-1^+^) or neurons (NeuN^+^) was assessed by immunofluorescence staining. Flow cytometry was used to detect the number and phenotype of microglia; the changes in microglial phagocytosis and the β-catenin signaling pathway were detected in BV2 cells overexpressing PKM2. For in vivo experiments, MRL/lpr mice were treated with AAV9-shPKM2. After 2 months, Morris water maze and conditional fear tests were applied to investigate the cognitive ability of mice; H&E and immunofluorescence staining were used to evaluate brain damage; flow cytometry was used to detect the phenotype and function of microglia; neuronal synapse damage was monitored by qRT-PCR, Western blotting, and immunofluorescence staining.

**Results:**

Glycolysis was elevated in the hippocampus of MRL/lpr lupus mice, accompanied by increased glucose consumption and lactate production. Furthermore, the activation of PKM2 in hippocampal microglia was observed in lupus mice. Cell experiments showed that PKM2 facilitated microglial activation and over-activated microglial phagocytosis via the β-catenin signaling pathway. In vivo, AAV9-shPKM2-treated mice showed decreased microglial activation and reduced neuronal synapses loss by blocking the β-catenin signaling pathway. Furthermore, the cognitive impairment and brain damage of MRL/lpr mice were significantly relieved after microglial PKM2 inhibition.

**Conclusion:**

These data indicate that microglial PKM2 have potential to become a novel therapeutic target for treating lupus encephalopathy.

**Supplementary Information:**

The online version contains supplementary material available at 10.1186/s12974-021-02279-9.

## Introduction

Systemic lupus erythematosus (SLE) is a complex autoimmune disease affecting various organs in the body [1, 2]. Clinically, 12–95% of SLE patients develop psychiatric and neurologic manifestations, also known as neuropsychiatric systemic lupus erythematosus (NPSLE), which severely affects life quality and increases disease-related mortality [3]. Among the 19 NPSLE syndromes defined by the American College of Rheumatology (ACR) [4], cognitive dysfunction has been identified as one of the most distressing symptoms in SLE [5]. It often develops insidiously, and presents and progresses independently. Also, it usually does not respond to standard immunosuppression [6, 7]. Accumulating evidence indicates that complex and interconnected mechanisms promote the development of SLE-related cognitive impairment; however, the specific pathogenesis is still unclear [3].

Brain function is correlated to an adequate metabolic cost, accounting for 20% of the whole-body energy consumption in humans [8, 9]. In 2000, positron emission tomography (PET) demonstrated that lupus cerebritis was positively associated with the significant alterations in glucose metabolism with regional specificity in the brains of SLE patients [10, 11]. Furthermore, an increase in total brain glutamine, glutamate, and lactate concentrations has been found in MRL/lpr lupus mouse model compared to the MRL^+/+^ control mice by ^1^H and ^13^C nuclear magnetic resonance (NMR) spectroscopy detection [12]. Moreover, metabolic changes were observed in specific brain regions in SLE patients with cognitive impairment by multimodal magnetic resonance imaging (MRI) [13]. Interestingly, aging brains showed high lactate levels and were susceptible to several neurodegenerative diseases accompanied by metabolic alterations, contributing to cognitive impairment [14]. In anti-DNA antibodies (DNRAbs)^+^ mice, significant metabolic changes and spatial memory impairment were observed with neuronal loss in hippocampal regions [15]. Taken together, these studies suggest that the regional metabolic abnormalities are critical neuropathological features on the cognitive manifestation in NPSLE.

Pyruvate kinase isoform M2 (PKM2), a crucial mediator of cellular energetics, catalyzes the conversion of phosphoenolpyruvate to pyruvate during glycolysis and is identified as the hub network protein for the regulation of idiopathic autism [10]. Furthermore, a proteomics-based approach revealed that PKM2 is one of the main differential proteins involved in the pathogenesis of infantile spasms with severe cognitive dysfunction [11]. Propofol-protective effects on ketamine-induced neonatal cognition damage have been linked to PKM2 expression in the hippocampus [16]. Moreover, PKM2 was found to be significantly oxidized in the hippocampi of mild cognitive impairment patients and functionally involved in energy metabolism and synaptic plasticity [17]. Besides, methamphetamine-induced neurocognitive deficits exhibited PKM2 activity impairment [18]. Nonetheless, PKM2 in cognitive dysfunction and progression of NPSLE remains poorly understood.

Microglia, the tissue-resident macrophages of the central nervous system (CNS), have a major role in brain homeostasis [19, 20]. In addition to their intended role in host defense, microglia continuously stretch, retract, and restructure to monitor the functional status of their surroundings and remove the accumulated metabolites or cell debris to maintain brain homeostasis [21–23]. In the adult brain, microglia regulate advanced cognitive functions, such as learning and memory [24, 25]. When homeostasis is altered, or the brain tissue structures are damaged, microglia undergo several dynamic changes, such as shortening and swelling of the cellular processes, changes in the surface phenotypes and secretory mediators, and increased proliferation response. These changes in cell morphology and function are known as “activated state” [26]. Microglia activation is a common pathological feature of a range of neurodegenerative diseases, including Alzheimer’s disease (AD) [27–29]. Also, microglia have a major role in NPSLE, and their abnormal activation has been detected in the hippocampus of several strains of lupus-prone mice (NZB/NZW, MRL/lpr, and FcγRIIB^−/−^Yaa) [30]. In the process of continuous inflammation, activated microglia-phagocytosed astrocytes promote neuronal apoptosis and aggravate depression index and cognitive dysfunction in mice with lupus [31–33]. Lupus antibodies, DNRAbs, directly activate microglia-mediated neuronal damage and impair cognitive performance [34]. Hence, in this study, we explored the role of microglia in the cognitive dysfunction of NPSLE mice. We analyzed and compared the metabolites in the hippocampal tissues of the validated neuropsychiatric lupus model and control mice. Based on these variations, we found that PKM2 might be the key molecule inducing NPSLE. We then explored SLE-mediated cognition and brain injury pathogenesis.

## Materials and methods

### Materials and reagents

Flow cytometry antibodies and reagents: FITC-conjugated anti-mouse CD11b, APC-conjugated anti-mouse CD45, PE-conjugated anti-mouse LAMP1 and PE-conjugated anti-mouse CD86 were purchased from Biolegend (USA), anti-CD16/CD32 Fc Block and myelin removal reads were purchased from Miltenyi Biotec (Germany). Western blot and immunofluorescence (IF) antibodies: IBA-1 (R&D systems, USA); VGLUT1 (Santa Cruz Biotechnology, USA); LAMP1 and CD68 were purchased from Abcam (United Kingdom); albumin, PSD95 and PKM2 were purchased from Proteintech (China). β-catenin, Cyclin-D1, c-Myc, ENO1, PFKFB3, HK1, LDHA, PDK1, GLUT1 were purchased from Cell Signaling Technology (USA), horseradish peroxidase (HRP)-linked goat anti-rabbit IgG (Fcmacs, China), NeuN (Santa Cruz Biotechnology), donkey anti-rat IgG H&L (Alexa Fluor® 647) (Abcam), goat anti-mouse IgG-TRITC (Abcam), rabbit anti-goat IgG-FITC (Fcmacs). ELISA kit: Maltose and Glucose assay kit (RayBiotech, USA), l-lactate assay kit (Cayman, USA), interleukin (IL)-6 (Fcmacs, China), IL-1β (Fcmacs, China). TRIzol reagent and SYBR green dye were bought from Invitrogen (Carlsbad, USA). PKM2 overexpression plasmid was purchased from Nanjing Jereh Company (China), and RFectPM Eukaryotic DNA Transfection Kit was purchased from Changzhou EMI Company (China). β-catenin protein inhibitor (KYA1797K) was purchased from MCE Company (USA).

### Adeno-associated virus (AAV) construction

The AAV used in this study was constructed as previously reported [35]. Briefly, AAV9 carrying the IBA-1 promoter driving the expression of shRNA targeting PKM2 (AAV9-IBA-1 promoter-shPKM2, indicated as AAV9-shPKM2). PKM2 shRNA was produced by Shanghai GeneChem Co., Ltd. (sense: CATGGTCCTGCTGGAGTTCGTG, antisense: CATTCTAGTTGTGGTTTGTCC).

### Animals and treatment

About 6- to 8-week-old female MRL/MpJ-Fas^lpr^ (MRL/lpr) and C57BL/6 (control) mice [36] were obtained from Shanghai Slaccas Laboratory Animal Breeding Company (Shanghai, China). All mice were maintained under specific pathogen-free (SPF) conditions at a 12 h light/dark cycle and 20–22 °C. The animals were allowed free access to drinking water. The mice were acclimatized to these housing conditions for at least one week. Sixty mice were randomly divided into the following groups: control (*n* = 20), MRL/lpr (*n* = 20), Vector (MRL/lpr + AAV9-vector, *n* = 10), shPKM2 (MRL/lpr + AAV9-shPKM2, *n* = 10). For AAV9 treatment groups, AAV9-shPKM2 or AAV9-vector were stereotactically injected into the lateral ventricle of MRL/lpr mice when MRL/lpr mice grown to 10-week-old. Viruses were diluted with phosphate-buffered saline (PBS), each mouse received an injection of 4 µL (5E10 vg) viruses. The intracerebroventricular (ICV) injection were performed perpendicular to the skull (*x* = 0.8 mm, *y* = 0.2 mm, *z* = 2 mm) using a microprocessor-controlled mini-pump, delivery was performed at a rate of 500 nL/min. After injection, the needle was left in place for 5 min prior to slowly retracting it from the ventricles. Afterward the mice were left underneath a warm light to recover their mobility. When MRL/lpr mice grown to week 20, the mice were sacrificed [37]. The experiments on mice were approved by Institutional Animal Care and Use Committee, Nanjing University, and all experiments were performed in accordance with relevant guidelines and regulations.

### Metabolites extraction

Hippocampus was dissected on a cold plate and frozen in liquid nitrogen. The tissue was homogenized in 80% methanol (made with methanol and water) on ice, vortexed for 30 s, sonicated for 10 min in an ice-water bath, and incubated for 1 h at − 20 °C to precipitate the proteins. The supernatant from the remaining sample was obtained by centrifugation at 12,000×*g* for 15 min at 4 °C. The extracts were dried in a vacuum concentrator without heating. A volume of 100 μL extraction solvent (V acetonitrile: V water = 1:1) was added for reconstitution, which was vortexed 30 s and sonicated for 10 min in a 4 °C water bath, followed by centrifugation for 15 min at 12,000×*g* at 4 °C. The supernatant (60 μL) of each sample was analyzed for metabolites [38].

### Metabolomic analysis by liquid chromatography–mass spectrometry (LC–MS)

LC–MS/MS analyses were performed using a UHPLC system (1290, Agilent Technologies Santa Clara, CA, USA) with a UPLC HSS T3 column (2.1 mm × 100 mm, 1.8 μm) coupled to Q Exactive (Orbitrap MS, Thermo, USA). The mobile phase A comprised 0.1% formic acid in water for normal phase (NP-HPLC) and 5 mmol/L ammonium acetate in water for reverse phase (RP-HPLC). The mobile phase B was acetonitrile. The elution gradient was set as follows: 0 min, 1% B; 1 min, 1% B; 8 min, 99% B; 10 min, 99% B; 10.1 min, 1% B; 12 min, 1% B. The flow rate was 0.5 mL/min, and the injection volume was 2 μL. A QE mass spectrometer was used to record MS/MS spectra in an information-dependent manner during the LC–MS experiment. In this mode, acquisition software (Xcalibur 4.0.27, Thermo) continuously evaluates the full-scan survey MS data as it collects and triggers MS/MS spectra acquisition based on the preselected criteria. The electrospray ionization (ESI) source conditions were set as follows: sheath gas flow rate of 45 Arb, Aux gas flow rate of 15 Arb, capillary temperature of 400 °C, full MS resolution of 70,000, MS/MS resolution of 17,500, collision energy of 20/40/60 eV in normalized collisional energy (NCE) model, and spray voltage of 4.0 kV (positive, POS) or − 3.6 kV (negative, NEG).

**Fig. 1:**
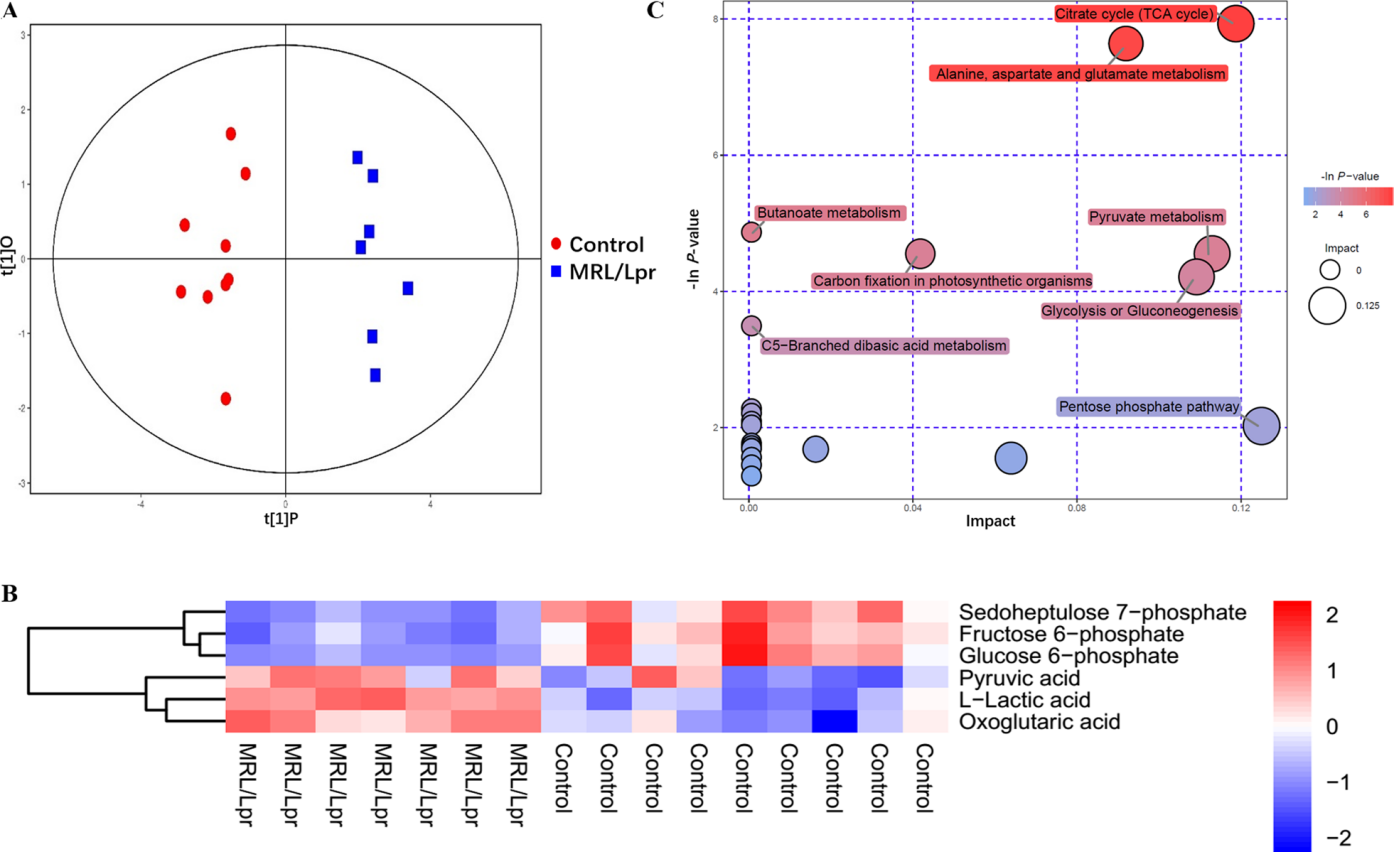
Central carbon metabolome analysis showing elevated metabolites in glycolysis in MRL/lpr mice hippocampus. **A** PCA model score scatter plot and OPLS-DA model showing separation of glycolysis metabolites in the hippocampus of control mice (blue) and MLR/lpr group mice (red). **B** Hierarchical clustering analysis for the significantly differential metabolites of LC–MS between the control and MLR/lpr groups of mice. The relative metabolite level was depicted according to the color scale: red indicates upregulation, whereas blue indicates downregulation. **C** Metabolomic map of significant metabolic pathways characterized in the hippocampus of control and MLR/lpr groups. The significantly altered pathways based on enrichment and topology analysis are shown in this metabolomic map. The *X*-axis represents pathway impact; *Y*-axis represents pathway enrichment. Large size and dark colors represent the major pathway enrichment and high pathway impact values, respectively

The raw data were converted into the mzXML format using ProteoWizard and processed by MAPS software (version 1.0). The preprocessing results generated a data matrix that consisted of retention time, mass-to-charge ratio (*m*/*z*), and peak intensity. An in-house tandem mass spectrometry (MS^2^) database was utilized for metabolite identification. The resulting three-dimensional (3D) data involving peak number, sample name, and normalized peak area were entered into SIMCA14.1 software package (V14.1, Sartorius Stedim Data Analytics AB, Umea, Sweden) for principal component analysis (PCA) and orthogonal projections to latent structure-discriminate analysis (OPLS-DA). PCA showed the distribution of the original data. Supervised OPLS-DA was applied to obtain a high level of group separation and identify variables responsible for classification. Sevenfold cross-validation was used to estimate the robustness and predictive ability of our model. The permutation test further validated the model. A loading plot was constructed on the basis of OPLS-DA and showed the contribution of variables to differences between the two groups. The first principal component of variable importance in projection (VIP) was obtained to refine the analysis. If *P* < 0.1 and *VIP* > 1, then the variable was defined as a significantly differential metabolite (SDM) between the groups. The SDMs, obtained from LC–MS, were imported into MetaboAnalyst 4.0 to explore different potential metabolic pathways in the hippocampus between MRL/lpr and control groups. As shown in Fig. 1C, the bubble plots demonstrated the main influential metabolic pathways [39].

### Measuring hippocampal glucose and lactate levels

The hippocampal tissues of different groups of mice were lysed to obtain hippocampal tissue homogenates. According to the manufacturer’s instructions, the Maltose and Glucose Assay Kit (RayBiotech, USA) was used to detect the glucose concentration in the hippocampal tissue homogenates, while the l-Lactate Assay Kit (Cayman, USA) was used to determine the concentration of lactic acid in the hippocampal tissue homogenate according to the manufacturer’s protocol. The glucose and lactate levels in mouse hippocampus and celebrate cortex were corrected for that of control group.

### Flow cytometry analysis of mouse microglia

The flow cytometry labeling and experimental method of mouse microglia were designed by Bennett et al. [40]. Six mice per group were used to flow cytometry detection. When the mice were killed, one side hippocampus/cortex was excised on ice immediately, and the tissue dispersed to prepare a single-cell suspension. According to the manufacturer’s instructions, the single-cell suspension was mixed with myelin removal beads, then automatic magnetic cell sorter was applied to remove myelin fragments from the single-cell suspension, so that the microglia could be enriched for labeling and detection by flow cytometry [39]. After sorting, single cells were pre-blocked with anti-CD16/CD32 Fc Block for 10 min, then stained on ice for 30 min with CD11b-FITC, CD45-APC and CD86-PE, CD86-PE or LAMP1-PE, rinsed twice with PBS, and finally resuspended in 200 μL buffer for subsequent evaluation by flow cytometry (BD Accuri™ C6, BD Biosciences, USA). Data were analyzed using FlowJo software. Microglia were defined as CD11b^+^CD45^lo^, proinflammatory microglia were defined as CD11b^+^CD45^lo^CD86^+^.

### Brain pathological evaluation

Four mice per group were used for pathological evaluation. The whole-brain of the mice was fixed with 4% polyoxymethylene (PFA) and sliced into 5-mm-thick sections for hematoxylin/eosin (H&E) staining.

### PKM2 detection in microglia and neurons by immunofluorescence (IF) staining

Double IF staining was carried out to detect the expression of PKM2 in microglia and neurons in hippocampus tissue. Briefly, 8-µm-thick frozen brain slices were fixed in cold methanol/acetone (1:1) for 10 min at − 20 °C. After PBS washes, the samples were blocked with 3% bovine serum albumin (BSA) in PBS for 60 min at room temperature, followed by incubation with anti-IBA-1, anti-PKM2, and anti-NeuN primary antibodies (1:200) overnight at 4 °C. Subsequently, the samples were incubated with goat anti-mouse IgG-TRITC and rabbit anti-goat IgG-FITC secondary antibodies (1:400) for 1 h at room temperature in the dark, followed by nuclei staining with 2-(4-amidinophenyl)-1H-indole-6-carboxamidine (DAPI).

To evaluate the neuronal degeneration, double staining of NeuN and the TUNEL was performed using the In Situ Cell Death Detection Kit (Roche Diagnostics) [41]. Briefly, double staining of NeuN and the TUNEL was performed using the In Situ Cell Death Detection Kit (Roche Diagnostics). After immunohistochemical staining of IBA-1 as described above, the sections were washed three times in PBS and then incubated with the TUNEL solution containing FITC-dUTP for 60 min at 37 °C. Finally, the sections were washed in PBS and mounted for the analysis. All sections were examined by a Nikon Eclipse Ti-U fluorescence confocal microscope, which equipped with a digital camera (FV300, Olympus, Japan).

### RNA extraction and quantitative real-time PCR (qPCR)

Six mice per group were used to assess the expression of mRNA. Total RNA was extracted from cells or tissues using TRIzol reagent. According to the manufacturer’s instructions, 1 μg total RNA was reverse transcribed in a 20-μL reaction system. The oligonucleotide primers used for PCR amplification are listed in Table 1. All reactions were carried out in triplicate. The expression levels of the target genes were normalized to that of *GAPDH*.

**Table 1:** Primers used for q-PCR in this study

Gene	Forward	Reverse
*GAPDH*	GCATGGCCTTCCGTGTTCC	GGGTGGTCCAGGGTTTCTTACTC
*PKM2*	TCAGAGCTCCAACGCTTGTAGAACTCACTC	CCGCTCGAGAAATGGAAGGTGGAGGG
*ENO1*	GCCTCCTGCTCAAAGTCAAC	AACGATGAGACACCATGACG
*HK1*	TGCCATGCGGCTCTCTGATG	CTTGACGGAGGCCGTTGGGTT
*PFKFB3*	ATTGCGGTTTTCGATGCCAC	GCCACAACTGTAGGGTCGT
*LDHA*	ATGGCAACTCTAAAGGATCA	GCAACTTGCAGTTCGGGC
*PDK1*	AGGCAAAGGAAGTCCATCTCA	CCCATGCATTTGTGCCTACC
*GLUT1*	CAATGCTGATGATGAACCTGCTGG	GAACACCTGGGCGATGAGGATG

### Western blot

Proteins of one side hippocampus/cortex were extracted by standard techniques [42]. Four mice per group were used for Western blot detection. Typically, total proteins were separated by sodium dodecyl sulfate-polyacrylamide gel electrophoresis (SDS-PAGE), then the total protein were transferred to polyvinylidene fluoride (PVDF) membranes (Millipore Co, Bedford, MA, USA). The membranes were blocked in 5% BSA dissolved in TBST (50 mM Tris/HCL, pH 7.6, 150 mM NaCl and 0.1% Tween-20) for 2 h at room temperature and probed with indicated primary antibodies overnight at 4 °C, washed the membrane three times with TBST, followed by incubation with appropriate HRP-linked secondary antibody 2 h at room temperature, washed the membrane three times. The immunoreactive bands were visualized using enhanced chemiluminescence (ECL) plus Western blot detection reagents (Supersignal™ West Pico PLUS, Thermo, USA).

For the membranes that needs to be stripped of antibodies, the membranes were washed with TBS three times, incubated with restore Western blot stripping buffer (Thermo scientific, USA) in the dark at 37 °C for 20 min, washed with TBS 3 times [43, 44]. Then, the membranes were re-blocked in 5% BSA for 30–60 min at room temperature, and probed with indicated primary antibodies overnight at 4 °C, subsequent steps are the same as conventional WB. The gray values were analyzed by Image J software (National Institutes of Health, Bethesda, MD, USA).

### Cytokine detection by enzyme-linked immunosorbent assay (ELISA)

Four mice per group were used for ELISA detection. ELISA was used to detect the content of inflammatory factors (IL-1β and IL-6) in the hippocampus of mice using the commercial kits, according to the manufacturer’s instructions. The mouse hippocampal tissues protein extract was at dilution of 1:5.

### Cell culture and treatment

Microglia cell line BV2 cells and neuronal cell line HT22 cells were generous gifts from Prof. Tianjiao Xia, Nanjing University and cultured in Dulbecco’s modified eagle’s medium (DMEM) containing 10% fetal bovine serum (FBS; Gibco) at 37 °C with 5% CO_2_. 2 × 10^5^ BV2 cells/mL was seeded in 6-well, when cell confluency exceeded 30%, the cells were transfected with PKM2 overexpression plasmid. The cells were treated with β-catenin inhibitor (KYA1797K), at a 200 ng/mL concentration. After that, BV2 cells were co-cultivated with HT22 cells at a ratio of 1:3 for 24 h and collected cells for subsequent experiments.

### Detection of microglial phagocytosis

The phagocytic capacity of microglia was determined using the method described previously [45]. Briefly, BV2 cells were seeded into 6-well plates at a density of 2 × 10^5^ cells/well in DMEM medium. The fluorescent yellow-green labeled 1-μm amine-modified polystyrene latex beads were mixed with FBS at a ratio of 1:5 and incubated in a 37 °C water bath for 1 h, then this mixture was diluted with DMEM complete medium to achieve the final concentration of fluorescent beads and FBS in DMEM as 0.01% (v/v) and 0.05% (v/v), respectively. Subsequently, the cells were washed twice with PBS and incubated with DMEM, containing fluorescent beads, for 1 h at 37 °C. Finally, the cells were collected for flow cytometry detection.

### Behavioral assays

Ten mice per group were used to carry out behavioral detection. The majority of the 16-week-old MRL/lpr mice exhibited characteristic cognitive dysfunctions [46]. Morris water maze (MWM) test was conducted to assess hippocampus-dependent spatial learning and memory functions in rodents [47, 48]. The MWM tests included two parts: the spatial acquisition and probe trials. The spatial acquisition trial was performed for five consecutive days. In each trial, the mouse was allowed to search the platform within 60 s, and could stay on the platform for 5 s after it was located. The mouse that failed to find the platform in 60 s was guided to it manually and ordered to remain on the platform for 15 s, which was regarded as latency. The time spent on searching and mounting the platform (latency) was calculated. On day 6, a probe trial was performed for reference memory by removing the platform. The mice were randomly placed into two selected quadrants, which had different distances to the platform and were allowed to swim freely for 60 s. The percentage of time spent in the target quadrant and platform crossings was recorded, analyzed, and considered as an indicator of memory retention.

The fear-conditioning paradigm was also assessed [49, 50]. Briefly, mice were trained to associate cage context or an audiovisual cue with an aversive stimulus (foot-shock). The test was administered over 2 days. On day 1, mice were placed in a cage and exposed to two periods of 30 s each of paired cue light and 3000-Hz tone, followed by a 2-s foot-shock (0.8 mA) with a 180-s interval. On day 2, mice were subjected to two trials. In the first trial assessing contextual memory, mice were re-exposed to the same cage context, and freezing behavior was measured over 1–3 min, using a FreezeScan tracking system (CleverSys, Inc., Reston, VA). In the second trial measuring cued memory, mice were placed in a novel context and exposed to the same cue light and tone from day 1 after 2 min of exploration. The freezing behavior was measured for 1–3 min following the cue.

### Statistical analysis

All data are presented as mean ± standard error of mean (SEM), and each experiment included triplicate sets. The significant differences among groups with one independent variable were determined by one-way analysis of variance (ANOVA) with a Tukey’s multiple comparisons test for planned comparisons. *P*-value ≤ 0.05 was considered significant. GraphPad Prism 5 was used for data analysis (GraphPad Software Inc., CA, USA).

## Results

### Central carbon metabolome analysis reveals that elevated glycolysis is involved in hippocampal tissues in MRL/lpr lupus mice

The hippocampus has an important part in the pathogenesis of NPSLE [51, 52]. To understand the alterations on central carbon metabolism in the hippocampus of neuropsychiatric lupus mice, the hippocampus from 20-week-old female MRL/lpr and control mice was harvested for targeting metabolite analysis. Eleven metabolites, including 6-phosphogluconic acid, were differentially expressed in the two groups (Table 2).

**Table 2:** Metabolic overall analysis among the NPSLE model group and control group

Metabolite name	Log-fold change	*P*-value	VIP	*Q*-value
6-Phosphogluconic acid	6.176E−03	0.986	0.346	0.329
d-Glucose	9.690E−02	0.176	0.528	0.080
d-Ribulose-5-phosphate	4.066E−01	0.03	1	0.017
Fructose 6-phosphate	− 1.717E+00	4.312E−04	1.325	5.190E−04
Glucose 6-phosphate	− 2.832E+00	0.001	1.378	6.017E−04
l-Lactic acid	3.554E−01	9.050E−06	1.474	3.301E-05
Malic acid	6.597E−03	0.964	0.148	0.324
Oxoglutaric acid	3.296E−01	0.026	0.829	0.016
Pyruvic acid	7.167E−01	0.038	0.855	0.020
Sedoheptulose 7-phosphate	− 1.841E+00	1.881E−04	1.414	3.430E−04
Succinic acid	− 2.718E−01	0.261	0.580	0.115

Based on PCA and OPLS-DA analysis, a significant difference in the metabolites of the hippocampus were observed between MRL/lpr and control mice (Fig. 1A, B Additional file 1: S1A, S1B), and all the samples were within the 95% confidence interval (Hotelling’s T-squared ellipse). Briefly, the levels of pyruvic acid, l-lactic acid, and oxoglutaric acid (*P* ≤ 0.05) were significantly elevated in the hippocampus of MRL/lpr mice, while sedoheptulose 7-phosphate, fructose 6-phosphate, and glucose 6-phosphate (*P* ≤ 0.05) were significantly decreased (Fig. 1B). In addition, signaling pathway analysis revealed elevated glycolysis and pyruvate in hippocampal tissues in MRL/lpr mice (Fig. 1C), indicating that the glycolysis metabolic pathway in the hippocampus of lupus mice was significantly altered.

### NPSLE induces increases in glucose consumption and lactic acid production in the hippocampus

**Fig. 2:**
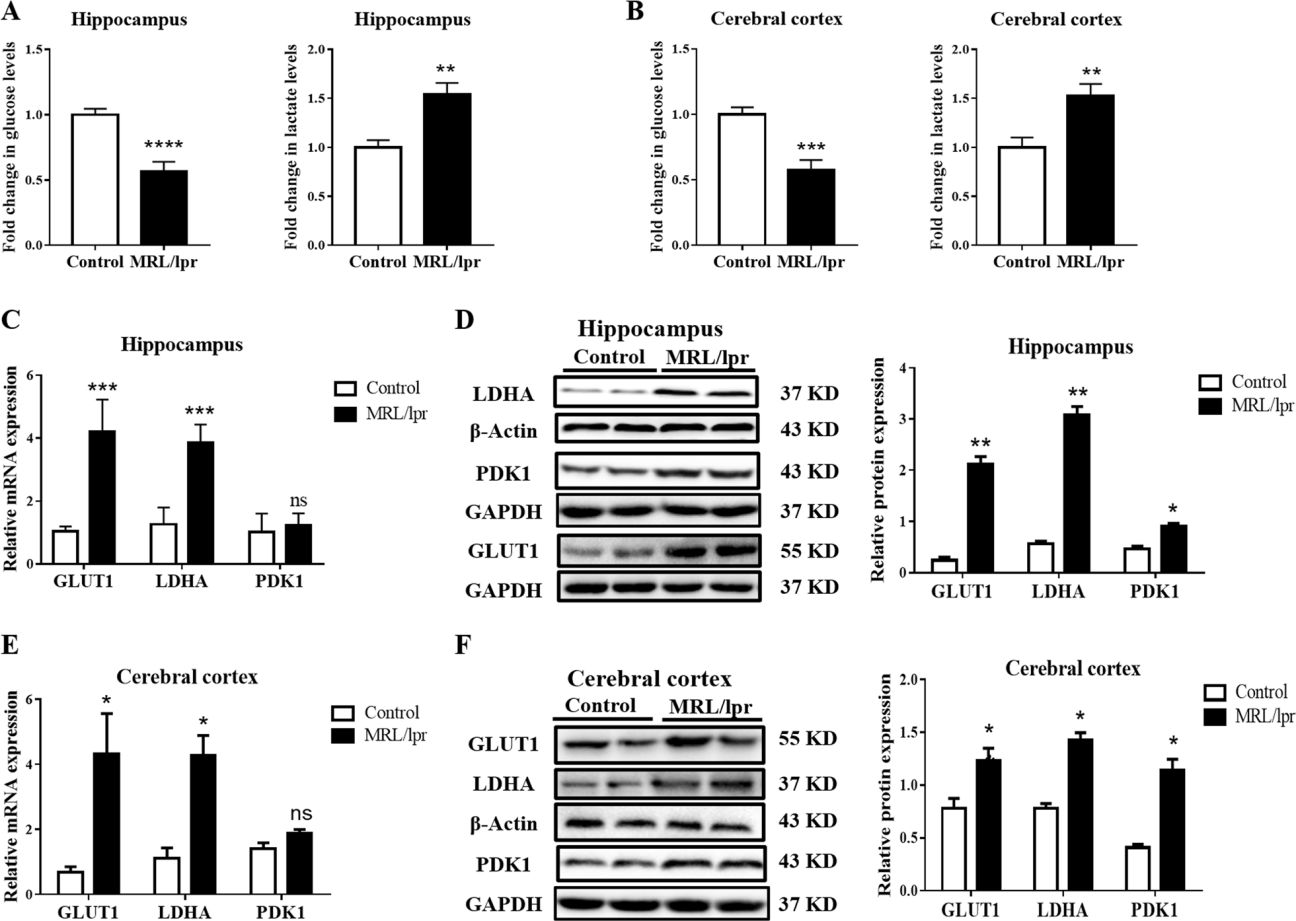
NPSLE promotes cell glycolysis in the hippocampus, with increased expression of GLUT1, PDK1, and LDHA. **A** Glucose levels and lactate production in the hippocampus of the MLR/lpr mice were normalized to that of the control group mice, *n* = 6. **B** Glucose levels and lactate production in the cerebral cortex of the MLR/lpr mice were normalized to that of the control group mice, *n* = 6. **C** The mRNA expression of *GLUT1*, *LDHA*, and *PDK1* in the hippocampus of control and MLR/lpr groups, *n* = 6. **D** Western blot quantification of GLUT1, LDHA, and PDK1 in the hippocampus of control and MLR/lpr groups, *n* = 4. **E** The mRNA expression of *GLUT1*, *LDHA*, and *PDK1* in the cerebral cortex of the control and MLR/lpr groups, *n* = 6. **F** Western blot quantification of GLUT1, LDHA, and PDK1 in the cerebral cortex of control and MLR/lpr groups, *n* = 4. Data are presented as mean scores ± SEM, *P ≤ 0.05, **P ≤ 0.01, ***P ≤ 0.001

Based on the metabolic profiles in the lupus hippocampus, we further detected glucose consumption and the production of lactic acid in the hippocampal tissues via ELISA. Compared to the control group, the glucose consumption in the NPSLE group significantly increased (about twofold decreased, *P* ≤ 0.005, Fig. 2A), accompanied with the increase lactate production (about 1.5-fold increased, *P* ≤ 0.01, Fig. 2A); the same was also observed in the cortical tissues (Fig. 2B). Furthermore, we built another murine model of lupus induced by imiquimod (IMQ) [53]. In the hippocampus and cerebral cortex of the IMQ group, the glucose consumption and lactate production were significantly increased, which was consistent with the results from the typical NPSLE mice model (*P* ≤ 0.01, Additional file 1: Fig. S2A). Notably, glucose was transported across the plasma membrane by glucose transporters (GLUT); the mRNA and protein expression of GLUT1 (4.2-fold and sixfold increase, respectively, *P* ≤ 0.05) were significantly upregulated in the hippocampus of lupus mice compared to control mice (Additional file 1: Fig. S2B–D). The mRNA and protein levels of hippocampal lactic dehydrogenase kinase A (LDHA) and pyruvate dehydrogenase kinase 1 (PDK1) (sixfold and twofold increase, respectively, *P* ≤ 0.05) were also upregulated (Fig. 2C, D). Cortical GLUT1, LDHA, and PDK1 levels were upregulated, except for the *PDK1* gene (*P* ≤ 0.05, Fig. 2E, F and Additional file 1: Fig. S2C).

To sum up, these results indicated that the glycolysis was altered in the hippocampus of NPSLE mice, showing increased glucose consumption and lactic acid production.

### Glycolysis promotes PKM2 activation in hippocampal microglia

**Fig. 3:**
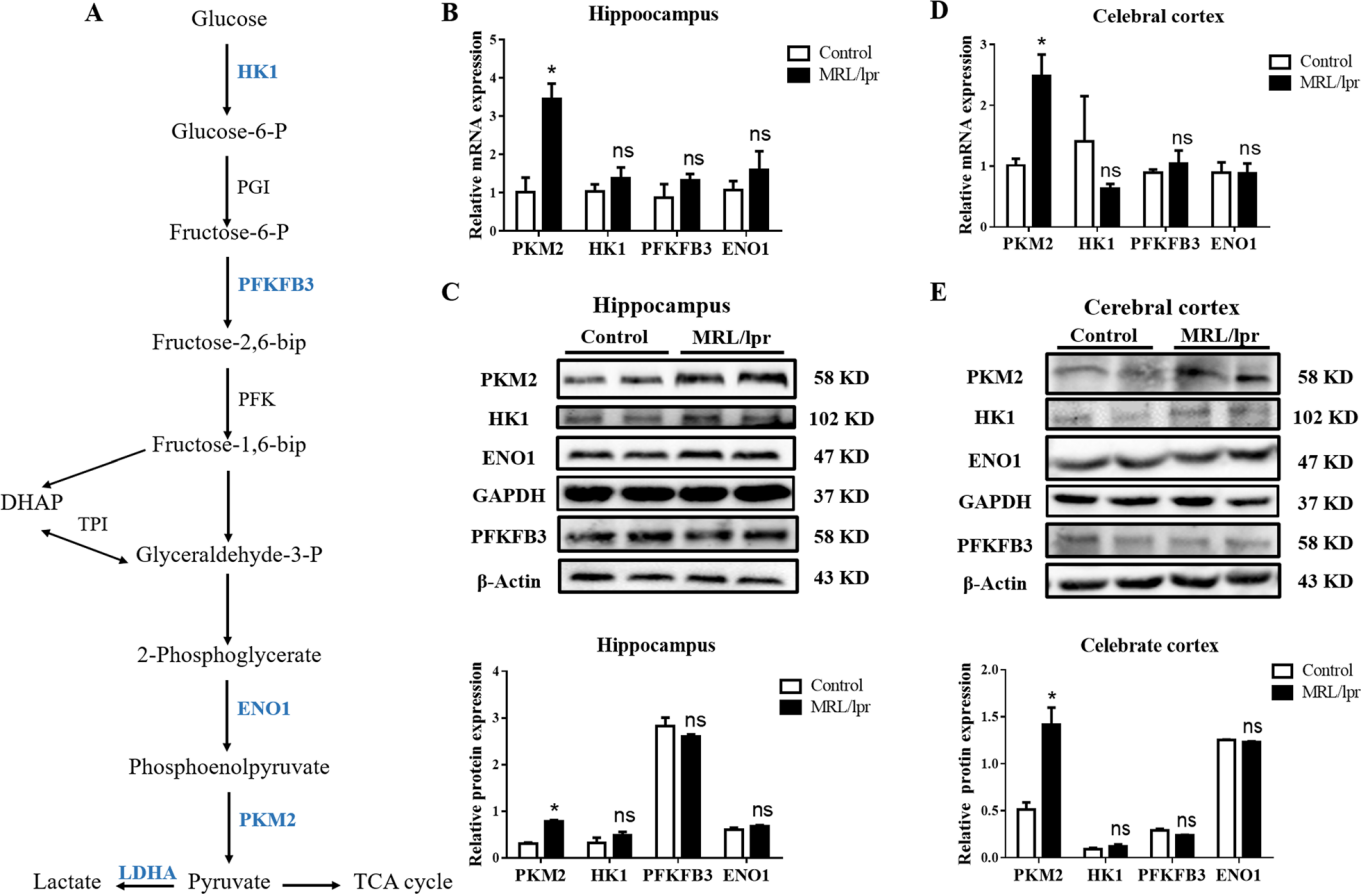
Glycolytic enzyme PKM2 is upregulated in NPSLE mice. **A** The simplified scheme indicating the main metabolic routes followed by glucose, the key intermediates and enzymes involved, and the enzymes studied in the text are highlighted for simplicity. Dashed arrows indicate downstream intermediates or metabolic pathways. **B** The mRNA expression of *PKM2*, *HK1*, *PFKFB3*, and *ENO1* in the hippocampus of control and MLR/lpr groups, *n* = 6. **C** Western blot quantification of PKM2, HK1, PFKFB3, and ENO1 proteins in the hippocampus of control and MLR/lpr groups, *n* = 4. **D** The mRNA expression of *PKM2*, *HK1*, *PFKFB3*, and *ENO1* in the cerebral cortex of control and MLR/lpr groups, *n* = 6. **E** Western blot quantification of PKM2, HK1, PFKFB3, and ENO1 in the cerebral cortex of control and MLR/lpr groups, *n* = 4. Data are presented as mean scores ± SEM, *P ≤ 0.05

Based on the glycolysis pathway (Fig. 3A), we detected the expression of several critical glycolytic enzymes (HK1, PFKFB3, ENO1, and PKM2) in the hippocampus. Interestingly, only PKM2 expression was significantly increased (3.2-fold increase, *P* ≤ 0.05), while the other key enzymes remained unchanged (Fig. 3B, C, and Additional file 1: Fig. S3A); similar results were observed in cortical tissue (Fig. 3D, E and Additional file 1: Fig. S3B). These data suggested that the abnormal metabolism of glycolysis in the brain of NPSLE mice could be attributed to the upregulation of PKM2.

**Fig. 4:**
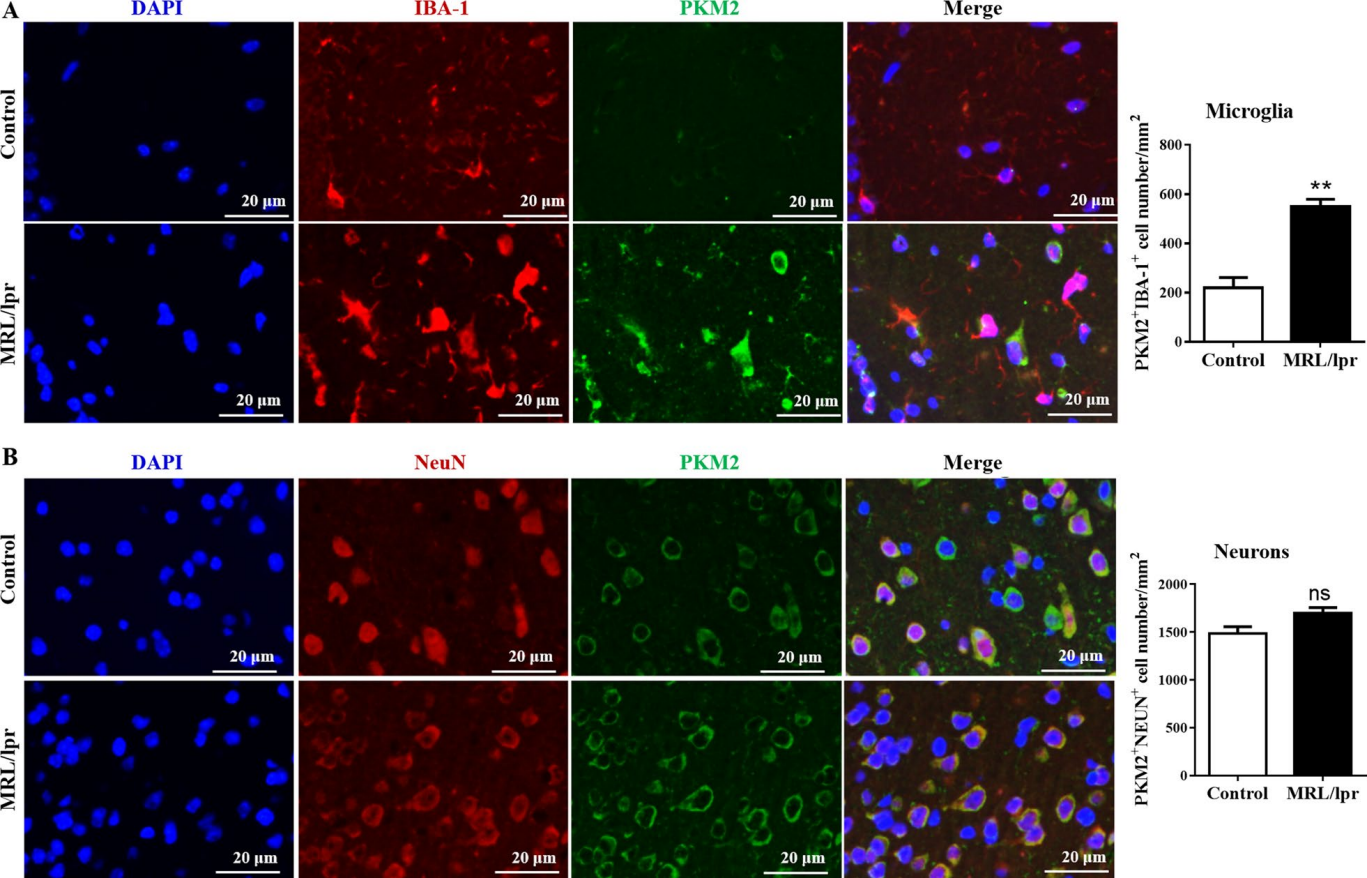
The colocalization of PKM2 with microglia increases in the hippocampus of MRL/lpr mice. **A** The expression levels of IBA-1 (red) and PKM2 (green) and their colocalization (yellow) in the hippocampus of control and MLR/lpr groups mice were detected by IF staining; Bar = 20 μm, *n* = 4. **B** The expression levels of NEUN (red) and PKM2 (green) and their colocalization (yellow) in the hippocampus of control and MLR/lpr groups mice detected by IF staining; Bar = 20 μm, *n* = 4. Data represent the mean scores ± SEM, **P ≤ 0.01

To further explore in which cell types PKM2 was expressed, we analyzed the colocalization of PKM2 with microglia (IBA-1^+^) and neuron (NeuN^+^) by IF. Results showed that the colocalization of PKM2 with microglia in the hippocampus of MRL/lpr mice was significantly increased (mean 531 ± 43 cells/mm^2^) compared to the control mice (mean 233 ± 52 cells/mm^2^, *P* ≤ 0.01, Fig. 4A, B), which indicated that the abnormal glycolysis might be related to the upregulation of PKM2 in microglia.

As one of the key enzymes of glycolysis, PKM2 has a dimeric and a tetrameric form; however, the biological effects between the tetramer and the dimer were significantly different [54–57]. Our results showed that tetrameric formation of PKM2 (232 kDa) was enhanced in the hippocampus of MRL/lpr mice compared to control mice (Additional file 1: Fig. S4A), which was consistent with the results of glucose consumption in the hippocampus of MRL/lpr mice. Simultaneously, we observed that the level of PKM2 dimer (116 kDa) in the hippocampus of MRL/lpr mice was increased (Additional file 1: Fig. S4B), which suggested that the dimer PKM2 might enter the nuclear to regulate nuclear transcription.

In order to verify this inference, we stimulated BV2 microglia with Toll-like receptor7 (TLR7) agonist R848 in vitro to simulate the lupus environment. After R848 induction, total PKM2 in BV2 cells were over-expressed. Meanwhile, cytoplasmic PKM2 decreased, which suggested that PKM2 enters the nucleus more under R848 stimulation (Additional file 1: Fig. S4C). Similarly, the immunofluorescence staining intuitively confirmed that more PKM2 entered the nucleus after R848 treatment (Additional file 1: Fig. S4D).

These data indicated that the increased expression of dimer and tetramer PKM2 in the hippocampus of MRL/lpr mice might be involved in the occurrence and development of NPSLE.

### Increased microglial engulfment of neuronal synapses in the hippocampus of NPSLE mice

**Fig. 5:**
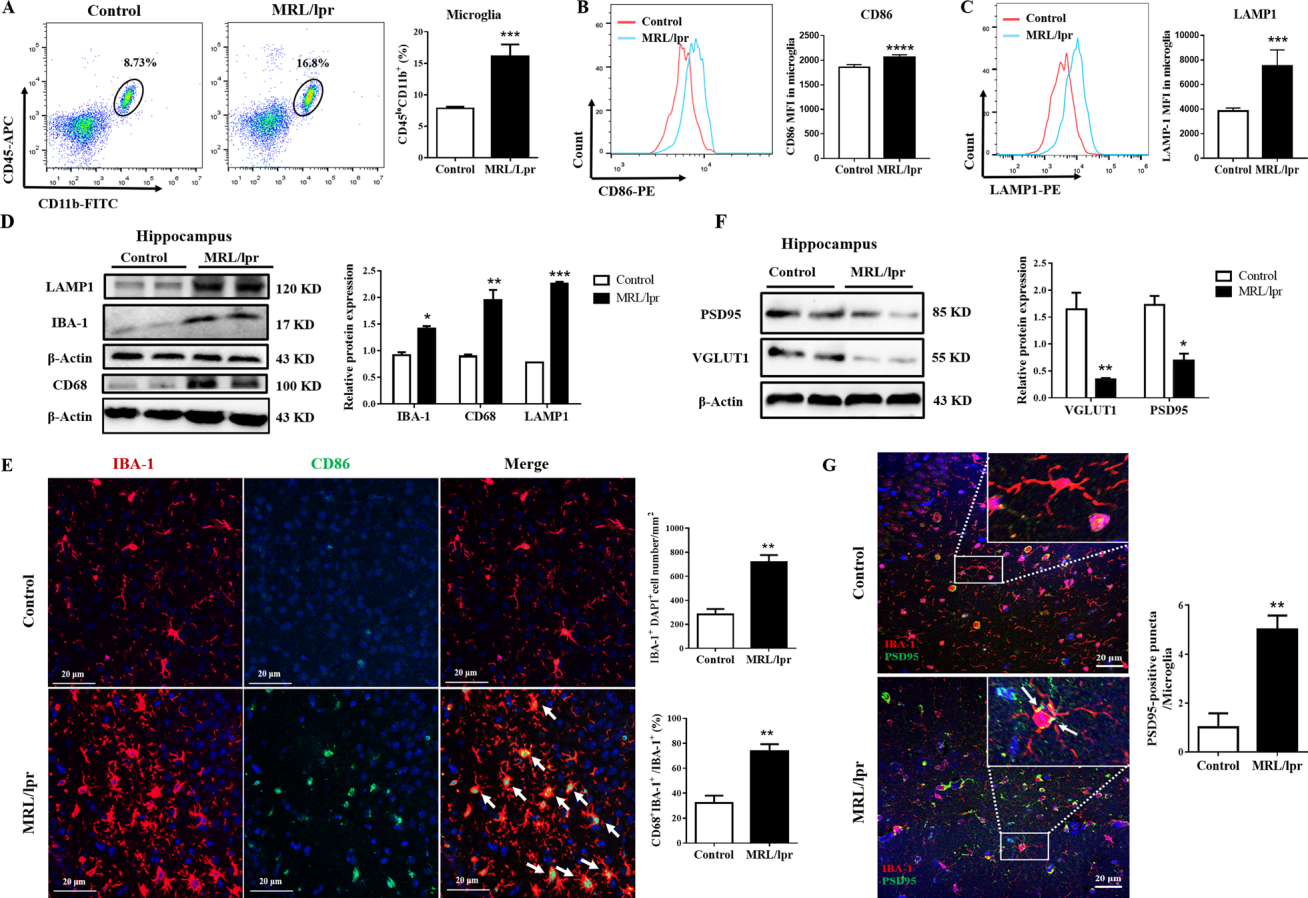
NPSLE activates microglia and induces the engulfment of neurons. **A** The percentage of microglia (CD45^lo^CD11b^+^) in the hippocampus of control and MLR/lpr groups, *n* = 6. **B** The MFI of CD86 on microglia of control and MLR/lpr groups was detected by flow cytometry, *n* = 6. **C** The MFI of LAMP1 in microglia of control and MLR/lpr groups was detected by flow cytometry, *n* = 6. **D** Western blot quantification of LAMP1, CD86, and IBA-1 in the hippocampus of control and MLR/lpr groups, *n* = 4. **E** The expression levels of CD68 (green), IBA-1 (red), and their colocalization (yellow) in the hippocampus of control and MLR/lpr groups were detected by IF staining; Bar = 20 μm, *n* = 4. **F** Western blot quantification of PSD95 and VGLUT1 in the hippocampus of control and MLR/lpr groups, *n* = 4. **J** The expression levels of PSD95 (green), IBA-1 (red), and their colocalization (yellow) in the hippocampus of control and MLR/lpr groups were detected by IF staining; Bar = 20 μm, *n* = 4. Data are presented as mean scores ± SEM, *P ≤ 0.05, **P ≤ 0.01, ***P ≤ 0.001, ****P ≤ 0.0001

To understand how microglia respond to the pathological PKM2 in the hippocampus of lupus mice, we calculated the percentage and phenotype of microglia in the hippocampus of two group mice by flow cytometry; the experimental procedure is shown in Additional file 1: Fig. S5A and B. Results showed that the microglia population (CD45^low^CD11b^+^) in MRL/lpr mice was significantly higher than that of control mice (16.1 ± 1.63% vs. 7.84 ± 0.23%, *P* ≤ 0.001, Fig. 5A). Besides, the expression of CD86 on microglia was upregulated in MRL/lpr lupus mice (median fluorescence intensity (MFI): 2062.87 ± 45.39 vs. 1858.37 ± 50.24, *P* ≤ 0.001, Fig. 5B). Apart from this, immunoblotting showed that the expression of IBA-1 and CD68 was obviously increased in the hippocampus of NPSLE mice compared to the control mice (Fig. 5D). In addition, the IF images of hippocampus confirmed that the population of IBA-1^+^ microglia cell was significantly increased (mean 28 cells/mm^2^ [CI 200–300 cells/mm^2^] vs. 716 cells/mm^2^ [CI 600–800 cells/ mm^2^] controls; *P* ≤ 0.01), with microglial thick cell bodies and abundant branches, and the expression of CD68 on IBA-1^+^ microglia was also elevated (Fig. 5E). Also, in the IMQ-induced lupus mouse model, microglial activation in the hippocampus increased markedly (Additional file 1: Fig. S6A, B, D, E). All these results suggested that hippocampus microglia were activated in lupus mice.

Abnormal phagocytosis of microglia has a major role in CNS and impairs cognitive functions such as learning and memory [58–60]. The expression of lysosome-associated membrane protein 1 (LAMP1) in the hippocampus was evidently upregulated, as shown by the Western blot results (*P* ≤ 0.001, Fig. 5D and Additional file 1: Fig. S6C). Meanwhile, flow cytometry detection showed that microglial LAMP1 also increased (MFI: 7499.2 ± 1176.38 vs. 1860.8 ± 210 controls, *P* ≤ 0.001, Fig. 5C). All above results indicated that the phagocytic function of hippocampal microglia in NPSLE mice was altered.

To further confirm our points, we detected VGLUT1 and PSD95 (neuronal synaptic markers) in the murine hippocampus. Compared to control mice, the expression of PSD95 (sevenfold decrease, *p* ≤ 0.05) and VGLUT1 (2.3-fold decrease, *P* ≤ 0.05) decreased in NPSLE mice (Fig. 5F), while the colocalization of PSD-95 and IBA-1 increased in MRL/lpr mice (Fig. 5G). After stimulation with R848, the phagocytosis of BV2 cells was monitored to assess microglia-mediated synapse engulfment. The results showed that the phagocytosis of BV2 cells increased after R848 stimulation (Additional file 1: Fig. S7A–D). Taken together, it could be deduced that the activation of microglia enhanced in the hippocampus of lupus mice promoted the purge of synapses by microglia.

### PKM2 facilitates microglial engulfment of neurons via β-catenin signaling

**Fig. 6:**
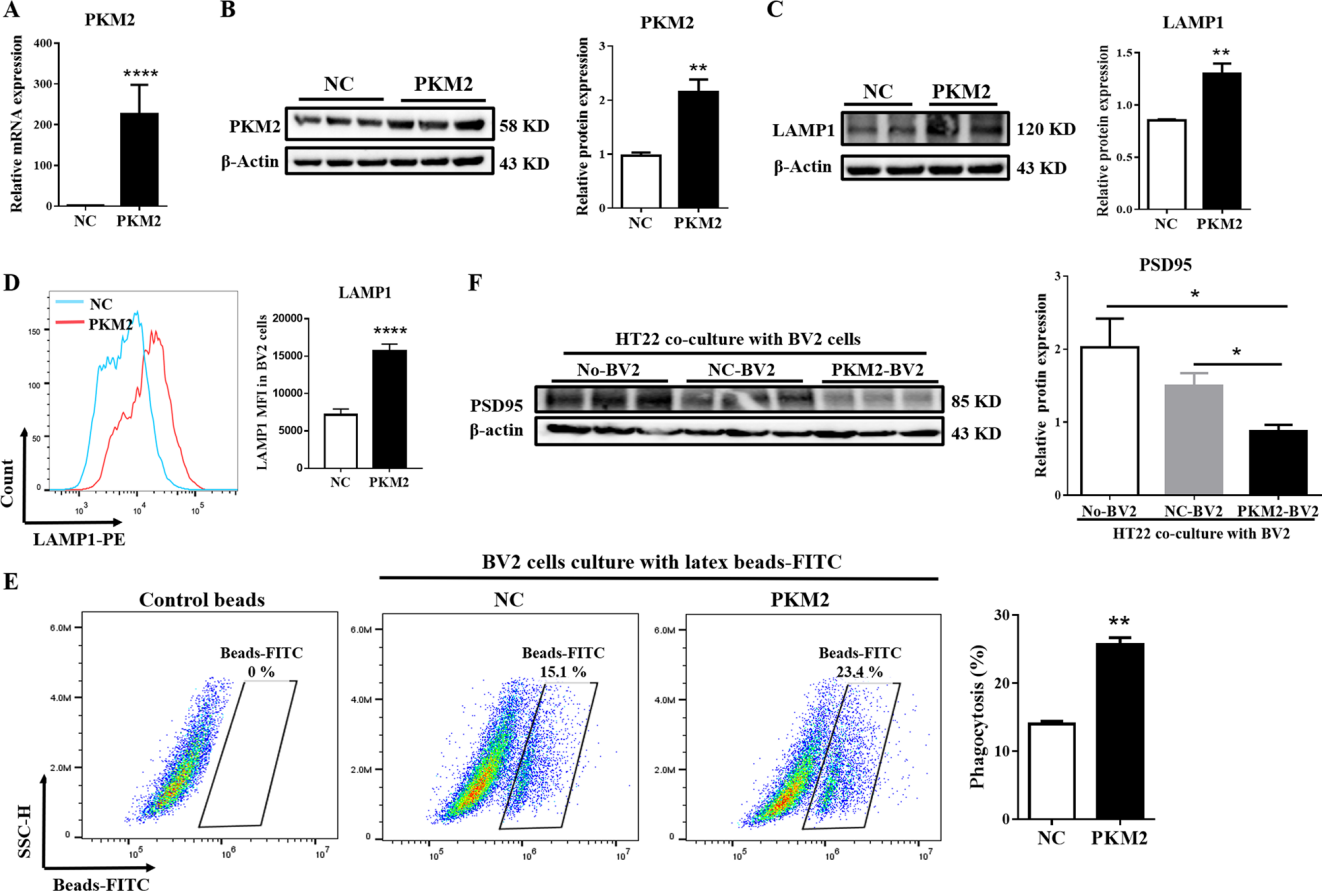
PKM2 overexpression stimulates phagocytic activity and engulfment of neurons. Transient transfection of BV2 cells with the negative control plasmid (NC) and PKM2 over-expressed plasmid (PKM2); 24 h later, the cells were analyzed for RT-PCR, Western blot, and flow cytometry. **A** The mRNA expression of *PKM2* was detected by RT-PCR. **B** Western blot quantification of PKM2 on BV2 cells. **C** Western blot quantification of LAMP1 on BV2 cells. **D** The expression level of LAMP1 detected by flow cytometry. **E** After BV2 cells were transfected with NC and PKM2 high expression plasmids, a phagocytic function test was performed, and the phagocytosis of BV2 cells were detected by flow cytometry. **F** After transfected with NC and PKM2 high expression plasmids, BV2 cells were co-cultured with HT22 cells, then Western blot was used to detect the protein level of PSD95. Data represent the mean scores ± SEM, *P ≤ 0.05, **P ≤ 0.01, ****P ≤ 0.0001. *n* = 3 per group

In vitro experiments showed that the increase in phagocytosis of BV2 cells was accompanied by a dose- and time-dependent increase in PKM2 after R848 stimulation (Additional file 1: Fig. S7F, G). In order to elucidate the correlation between PKM2 and microglial engulfment of neuronal structures, PKM2-overexpressing plasmids were transfected in BV2 microglia (*P* ≤ 0.05, Fig. 6A, B). Next, flow cytometry and Western blot experiments showed that the protein level of LAMP1 was significantly increased after PKM2 overexpression in BV2 cells (*P* ≤ 0.01, Fig. 6C, D). Fluorescent microsphere experiments proved that the phagocytosis of BV2 cells was significantly enhanced after overexpression of PKM2 (25.45 ± 0.81% vs. 13.57 ± 0.74% control; *P* ≤ 0.01, Fig. 6E). Moreover, a co-culture of BV2 and HT22 cells indicated that the expression of PSD95 on HT22 was downregulated in BV2 cells overexpressing PKM2 (Fig. 6F).

**Fig. 7:**
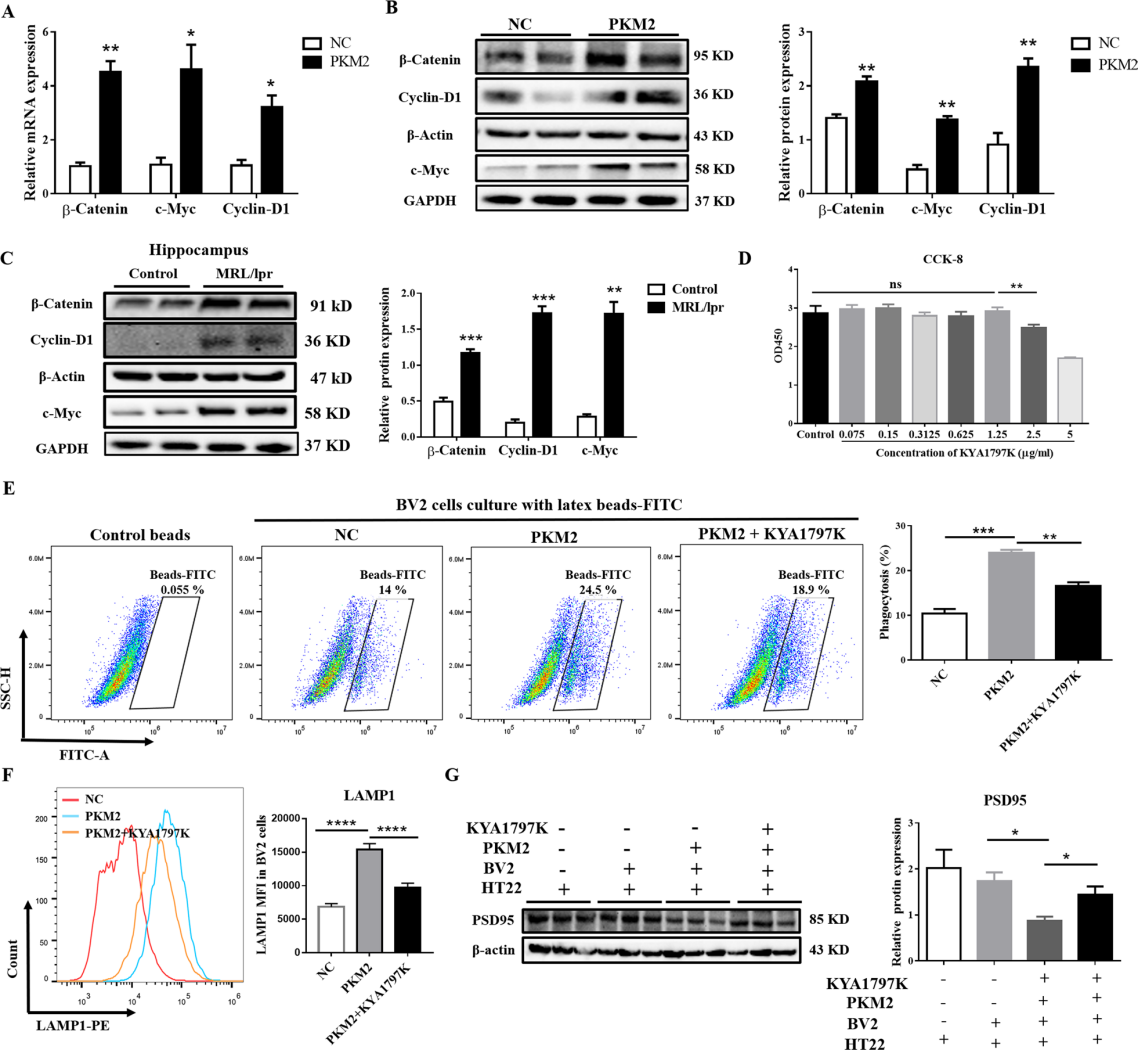
PKM2 facilitates phagocytosis via the β-catenin signaling pathway. **A** The mRNA expression levels of *β-catenin*, *c-Myc*, and *Cyclin-D1* detected by RT-PCR analysis at 24 h after plasmid transfection. **B** The protein expression of β-Catenin, c-Myc, and Cyclin-D1 detected by Western blot analysis 24 h after plasmid transfection. **C** Western blot quantification of β-catenin, c-Myc, and Cyclin-D1 in the hippocampus of control and MLP/lpr mice, *n* = 4. **D** BV2 cells were treated with different concentrations of β-catenin inhibitor (KYA1797K) for 24 h. CCK8 detected the cytotoxicity of KYA1797K to BV2 cells. **E** Transient transfection of BV2 cells with the negative control plasmid (NC) or PKM2 over-expressed plasmid (PKM2). After transfection, BV2 cells were treated with or without 200 ng/mL β-catenin inhibitor (KYA1797K) for 24 h. Then, a phagocytic function test was performed, and the phagocytosis of BV2 cells was detected by flow cytometry. **F** Transient transfection of BV2 cells with NC or PKM2 over-expressed plasmid, 24 h later, BV2 cells were treated with or without 200 ng/mL β-catenin inhibitor (KYA1797K) for 24 h. The expression level of LAMP1 was detected by flow cytometry. **G** BV2 cells were transiently transfected with NC or PKM2 over-expressed plasmid (PKM2); 24 h later, BV2 cells were treated with or without 200 ng/mL KYA1797K for 24 h, and then co-cultured with HT22 cells. Finally, a Western blot was used to analyze the protein level of PSD95. Data are presented as mean scores ± SEM, *P ≤ 0.05, **P ≤ 0.01, ***P ≤ 0.001, ****P ≤ 0.0001. *n* = 3 per group

The β-catenin signaling pathway is involved in the development of various CNS diseases [61–63]. Hence, we speculated that PKM2 upregulates microglial phagocytosis through the β-catenin signaling pathway. We detected the expression of the downstream molecular of the β-catenin signaling pathway on BV2 cells after PKM2 overexpression to confirm this speculation. Results showed that β-catenin and the downstream target genes/proteins c-Myc and Cyclin-D1 were upregulated after overexpression of PKM2 (*P* ≤ 0.05, Fig. 7A, B). In addition, these molecules also significantly increased after R848 stimulation (*P* ≤ 0.05, Additional file 1: Fig. S8A–D), and the mRNA levels of these genes showed a significant positive correlation with PKM2 (Additional file 1: Fig. S8A, B). Similarly, in vivo, the levels of β-catenin, c-Myc, and Cyclin-D1 were elevated in the hippocampus of NPSLE mice (*P* ≤ 0.01, Fig. 7C). These findings indicated that PKM2 could activate the β-catenin signaling pathway in vivo and in vitro.

To further demonstrate the involvement of the β-catenin signaling pathway in neuron and synapse loss in NPSLE mice, we selected KYA1797K to inhibit the expression of β-catenin [64]. The safe dose of KYA1797K was determined by the CCK8 experiment, and the final concentration of KYA1797K was selected to be 200 ng/mL for subsequent experiments (Fig. 7D). Then, fluorescent bead experiments showed that the enhanced microglial phagocytosis post-PKM2 overexpression was distinctly reversed after KYA1797K treated (*P* ≤ 0.05, Fig. 7E). Furthermore, the expression of LAMP1 in BV2 cells was downregulated (*P* ≤ 0.05, Fig. 7F), and the neuronal synapse phagocytosis of BV2 cells was weakened. Moreover, the application of KYA1797K in BV2 cells stimulated by R848 also showed similar results (Additional file 1: Fig. S8E–G). Based on these findings, we concluded that inhibit β-catenin signaling pathway reversed the PKM2-induced excessive phagocytosis of microglial cells.

### Microglial PKM2 inhibition alleviates brain damage and cognitive disorders in MRL/lpr mice

**Fig. 8:**
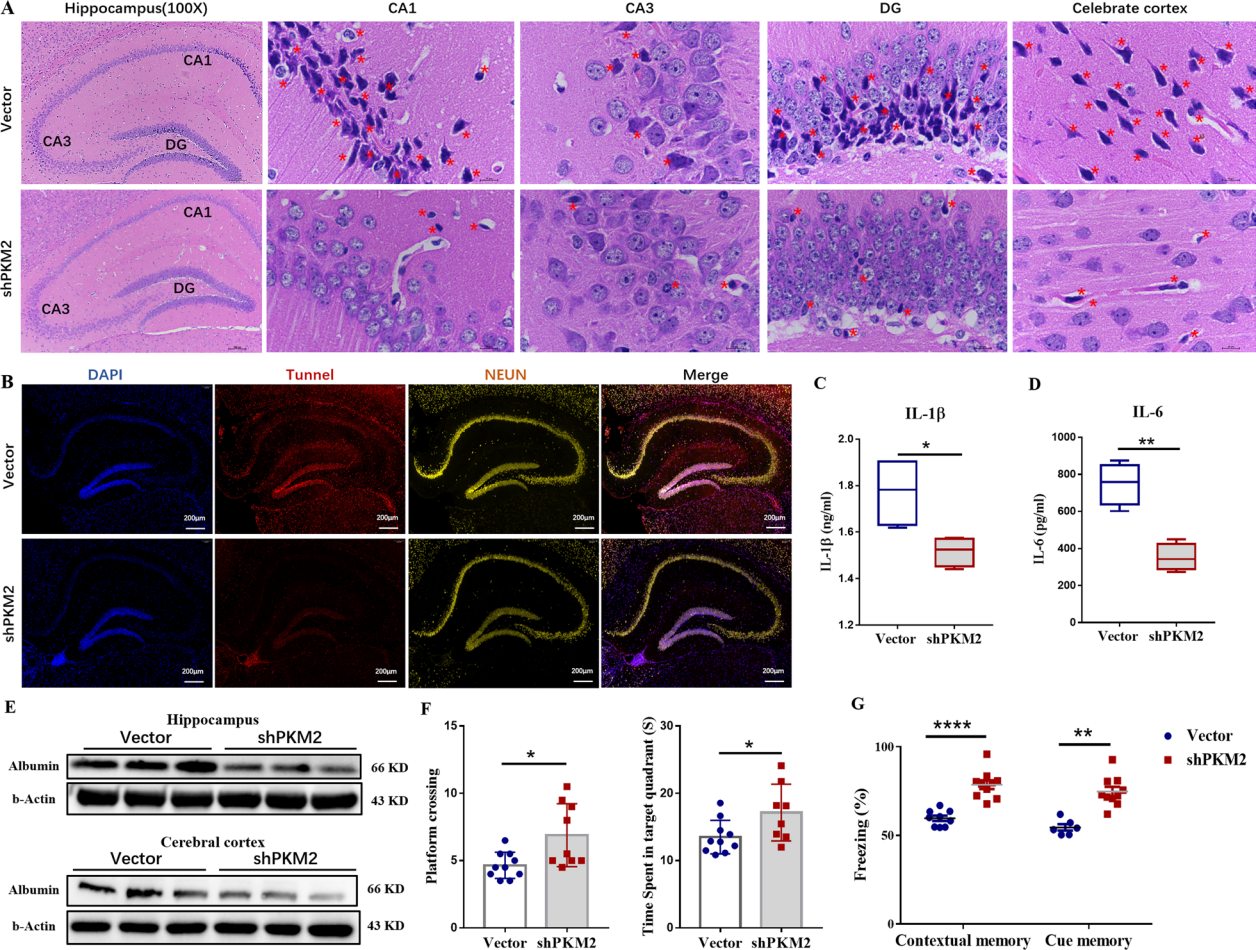
Microglial PKM2 inhibition alleviates brain damage and cognitive disorders in MRL/lpr mice. Twenty female MRL/lpr mice, aged 8 weeks, were divided into two groups: vector group and shPKM2 group. The mice were maintained until week 10. Empty vector virus or AAV9-shPKM2 virus were then stereotactically injected into the lateral ventricle of MRL/lpr mice, until the mice reached week 20. **A** HE staining of the coronal area of the mouse brain of each group mice; Magnification: Hippocampus (100 ×), CA1 (1000 ×), CA3 (1000 ×), DG (1000 ×), celebrate cortex (1000 ×), *n* = 4. **B** Tunel (red) and NeuN (yellow) immunofluorescence double staining of the hippocampus of each group mice; bar = 200 μm, *n* = 4. **C** The expression levels of IL-6 of the hippocampus of each group measured by ELISA, *n* = 6. **D** The expression levels of IL-1β in the hippocampus of each group measured by ELISA, *n* = 6. **E** Western blot quantification of albumin in the hippocampus and cortex, *n* = 4. **F** On day 6 of the water maze experiment, the time they stayed in the target quadrant (left) and the number of times the mice crossed the platform (right) was recorded, *n* = 10. **G** Fear conditioning tests. The percentage of the stiffness of mice was recorded during the test: different effects of 40-Hz light intervention, 2000-Hz sound intervention, and photoacoustic treatment on contextual and cue memory, respectively, *n* = 10. Data are represented as mean ± SEM, *P ≤ 0.05, **P ≤ 0.01, ****P ≤ 0.0001

Based on our above exploratory research, the AAV9-shPKM2 virus, targeting microglia (Additional file 1: Fig. S9A), was stereotactically injected into the lateral ventricle of MRL/lpr mice to specifically downregulate PKM2 on microglia. After we verified the accuracy of targeting interfering PKM2 (Additional file 1: Fig. S9B), the H&E staining showed that in microglial PKM2 inhibition group mice, the neurons in the hippocampal CA1, CA3, and DG brain regions were neatly arranged, the number of ghost cells was significantly reduced, and the injured neurons were significantly reduced (Fig. 8A). The neurophagy and microglial nodules were decreased in the cerebral cortex of the shPKM2 group compared to that of the empty vector group (Fig. 8A). Tunnel and NEUN co-staining determined injured neurons. The results showed that the injured neurons in the shPKM2 group were significantly reduced (Fig. 8B).

Neuroinflammation and albumin deposition in the CNS are usually regarded as pathological manifestations of NPSLE [65]. IL-1β and IL-6 are usually used as evaluation indicators of neuroinflammation in the hippocampus of MRL/lpr mice [66, 67]. Our results showed that the IL-6 and IL-1β cytokine levels in the hippocampus tissue were downregulated after microglial PKM2 inhibition (*P* ≤ 0.05, Fig. 8C, D). Meanwhile, fewer albumin deposits in the hippocampus were detected (Fig. 8E). In summary, these data suggest that microglial PKM2 inhibition might relieve the brain injury of NPSLE mice.

Subsequently, MWM and conditional fear tests were applied to investigate the cognitive ability in treated NPSLE mice. Regardless of the time mice took to cross the platform or stay in the quadrant, mice in the shPKM2 group showed better results than the vector group (Fig. 8F and Additional file 1: Fig. S10A-C). Similarly, the percentage of rigidity in environmental fear and fear of sound stimulation in the shPKM2 group were higher than in the vector group (Fig. 8G). Thus, we speculated that microglial PKM2 inhibition in NPSLE mice contributes in ameliorating learning and memory dysfunction.

**Fig. 9:**
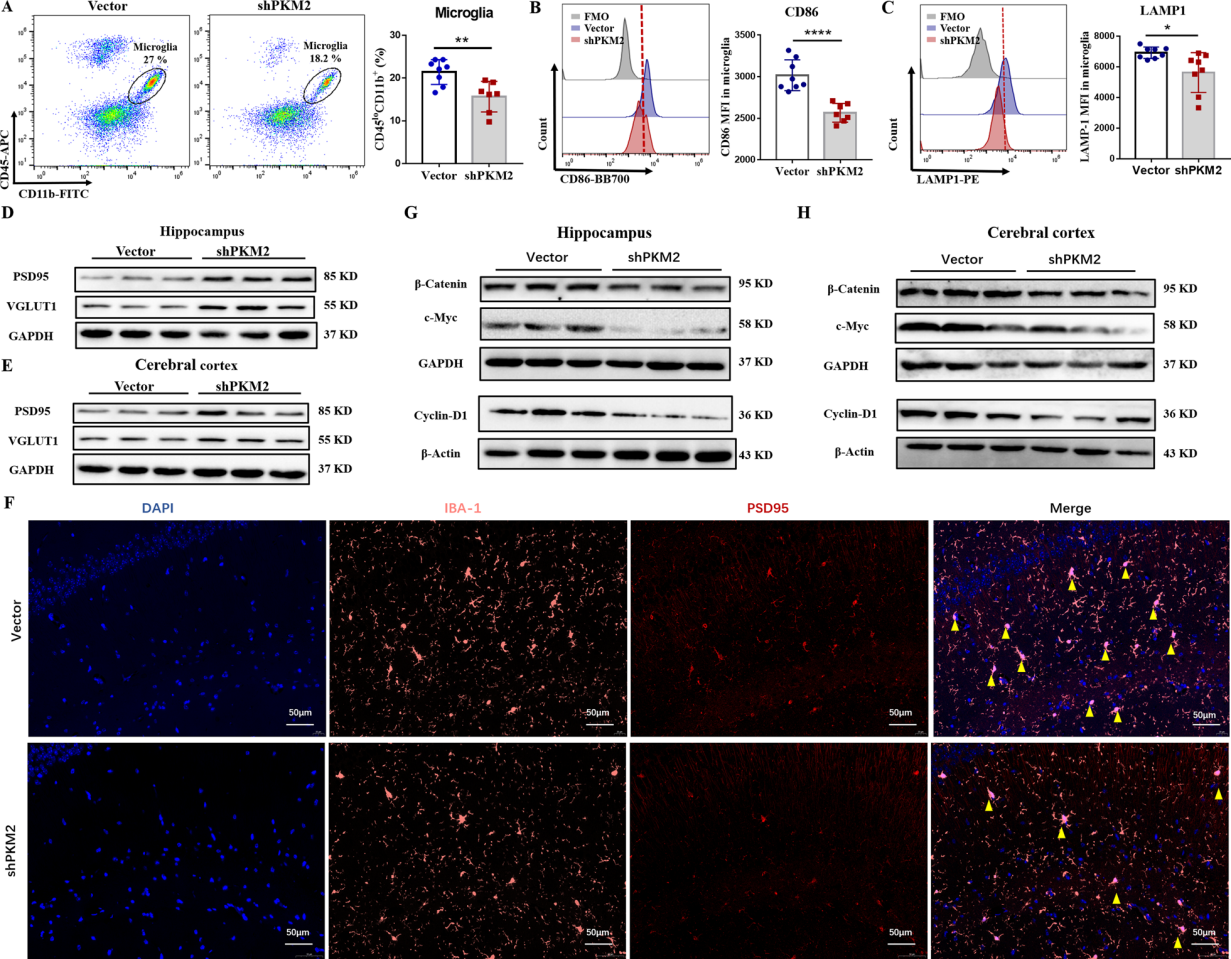
Silencing microglial PKM2 reduces microglial activation and loss of neuronal synapses by β-catenin signaling pathway. **A** The percentage of microglia (CD45^lo^CD11b^+^) in the hippocampus, *n* = 6. **B** The expression level of CD86 in microglia detected by flow cytometry, *n* = 6. **C** The expression level of LAMP1 in microglia detected by flow cytometry, *n* = 6. **D** Western blot quantification of PSD95 and VGLUT1 in the hippocampus of vector and sh-PKM2 group, *n* = 4. **E** Western blot quantification of PSD95 and VGLUT1 in the cerebral cortex of vector and sh-PKM2 group, *n* = 4. **F** PSD95 (red) and IBA-1 (pink) immunofluorescence double staining in the hippocampus of the mice in each group were detected by IF; Bar = 200 μm, *n* = 4. **G** Western blot quantification of β-catenin, c-Myc, and CyclinD1 in the hippocampus of vector and sh-PKM2, *n* = 4. **H** Western blot quantification of β-catenin, c-Myc, and CyclinD1 in the cerebral cortex of vector and sh-PKM2, *n* = 4. Data are represented as mean score ± SEM, *P ≤ 0.05, **P ≤ 0.01, ****P ≤ 0.0001

Furthermore, we observed that after silencing PKM2 on microglia, the number of hippocampal microglia was also decreased (*P* ≤ 0.01, Fig. 9A), while the proinflammatory phenotype CD86 and LAMP1 expression on microglia were downregulated (Fig. 9B, C). At the same time, PSD95 and VGLUT1 expression levels in the shPKM2 group mice were obviously upregulated, compared to the empty vector group (Fig. 9D, E). Immunofluorescence staining also showed less colocalization on IBA-1 (microglia marker) and PSD95 (neuronal synaptic marker) in the shPKM2 group (Fig. 9F). These data indicated that knocking-down PKM2 on microglia markedly suppresses the synaptic pruning of neurons in lupus mice. In addition, after microglial PKM2 inhibition, the protein levels of β-catenin, c-Myc, and Cyclin-D1 were significantly downregulated (Fig. 9G, H). In brief, this data suggested that microglial PKM2 inhibition alleviates cognitive disorders and brain damage in MRL/lpr mice and reduces microglial activation and loss of neuronal synapses by blocking the β-catenin signaling pathway.

## Discussion

This study showed that PKM2 was upregulated in lupus microglia with over-activated glycolysis in the hippocampus. PKM2 positively regulates the phagocytosis of microglia by the β-catenin signaling pathway, which aggravates the loss of neuronal synapses, and promotes the occurrence of cognitive dysfunction in NPSLE mice. Inhibition of the expression of microglial PKM2 could relieve the above symptoms. These results indicated that PKM2 might be a new target for the treatment of cognitive dysfunction in NPSLE.

Several factors, such as cerebrospinal fluid (CSF), IL-6 levels, and interferon-alpha (IFN-α), were recently reported to be closely related to the development of NPSLE [68]. In addition, autoantibodies and RNA–protein antigens form immune complexes in CSF and initiate the proinflammatory cascade [69]. However, the exact cause is not entirely clear. In patients with neuropsychiatric SLE, hippocampal structural lesions have been identified [51]. Similarly, structural abnormalities in the hippocampus of MRL/lpr mice have also been reported by Sakic et al*.* [52]. Unsurprisingly, our results revealed that IL-6 and IL-1β cytokine levels in the hippocampus of lupus mice were significantly higher than those in the control mice, with albumin accumulation in situ (data not shown). Besides, B cells chronically stimulated with B-cell activating factor, a cytokine associated with SLE, show enhanced glycolysis and subsequently synthesize more antibodies [70, 71]. Increased glycolysis has also been linked to mitochondrial oxidative stress in lupus-prone mice [72]. T cells exhibit persistent mitochondrial hyperpolarization due to increased mitochondrial reactive oxygen species production, depletion of reduced glutathione, and diminished mitochondrial ATP synthesis, which predisposes to proinflammatory death by necrosis [73]. These phenomena indicate that the metabolism reconfiguration might influence the pathogenesis of SLE [74, 75]. Interestingly, several neuroimaging reports indicated an association between cerebral hypometabolism and NPSLE [10, 11, 76], prompting us to further explore hippocampal metabolic abnormalities in MRL/lpr lupus mice.

In this study, we extracted the metabolites in the hippocampal tissues and conducted metabolomics analysis (LC–MS analysis), which exhibited abnormal glycolysis metabolites in the hippocampus of MRL/lpr mice. Under normal oxygen conditions, cells obtain energy via mitochondria oxidative phosphorylation and glycolysis. The activities and metabolic flux of the two pathways are delicately tuned to providing metabolic precursors for biosynthesis and energy production [77, 78]. Glucose consumption and lactic acid production in the hippocampus and cortex of MRL/lpr mice increase. Moreover, studies have indicated that active brain tissue does not fully oxidize glucose, but instead generates a local surplus of lactate, a phenomenon termed aerobic glycolysis [79, 80]. It remains to be clarified why is it important to engage in inefficient ATP production by glycolysis when energy demand is highest, and oxygen is plentiful. Substantial in vitro and in vivo evidence have indicated that neurons, the main energy consumers of brain tissue, could consume both glucose and lactate; the latter is produced by glycolysis [81–84]. Therefore, we speculated that even in the case of sufficient oxygen, there is an abnormal increase of aerobic glycolysis in the lupus brain to provide sufficient energy donors. Apart from this, in classically activated M1 macrophages, metabolism is shifted towards glycolysis. This switch increases glucose uptake and lactate production with activation of the pentose phosphate pathway and decreased mitochondrial oxygen consumption [80, 85, 86]. Microglia, the resident macrophages in the brain, revealed M1 polarization in the hippocampus of MRL/lpr mice, which might be one of the factors causing increased glucose consumption and lactate production in our results.

Glycolysis is the metabolic pathway that converts glucose into pyruvate, controlled by various glycolytic enzymes [87]. PKM2 is an important rate-limiting enzyme in glycolysis that catalyzes the irreversible conversion of phosphoenolpyruvate to pyruvate. This phenomenon could fuel the tricarboxylic acid cycle or convert pyruvate to lactate, which is then secreted [88]. PKM2 is highly expressed in cancer cells, activated immune cells [89, 90], and upregulated in most human cancers [91]. Over recent years, the regulatory role of PKM2 in autoimmune diseases has gained increasing attention. Previous studies have shown that pharmacological activation of PKM2 inhibits CD4^+^ T cell pathogenicity and suppresses experimental autoimmune encephalomyelitis [92]. PKM2-mediated aerobic glycolysis contributes to macrophage activation and inflammatory response, while PKM2 inhibitor protects the mice from lethal endotoxemia and sepsis [93]. Our current data indicated that PKM2 was abnormally expressed on the hippocampal microglia of MRL/lpr mice. However, in hypoxic-ischemic encephalopathy in neonatal rats, double IF labeling showed that PKM2 was mainly localized in the neurons of the ipsilateral cerebral cortex and not in astrocytes or microglia [94]. Some studies revealed that early recombinant PKM2 (rPKM2) treatment exerts an acute neuroprotective effect against ischemic brain damage, whereas delayed rPKM2 treatment promotes regenerative activities in the poststroke brain, thereby promoting functional recovery [95]. These phenomena suggested that PKM2 could be a target for the treatment of inflammatory brain diseases. Nevertheless, few studies have reported on the conformational differences of PKM2 in these inflammatory brain diseases.

It has been reported that PKM2 has both a dimeric form and a tetrameric form [53–55]. The biological effects between the tetramer and the dimer are significantly different [56, 57]. When PKM2 is in a tetrameric state, it has a higher affinity with its substrate phosphoenolpyruvate (PEP) and a higher PK enzymatic activity to catalyze the production of pyruvate by PEP [96–98]. After the tetramer is converted into a dimer, PKM2 can exist in a variety of different intracellular localization, and enter the nuclear to regulate gene expression [99]. PKM2 can also be modified with phosphorylation, acetylation, and other proteins to regulate protein activity and intracellular localization [100]. Our result showed that tetrameric and dimeric formation of PKM2 was enhanced in the hippocampus of MRL/lpr mice compared to control mice. Moreover, after R848 treatment, total PKM2 in BV2 cells were over-expressed while cytoplasmic PKM2 decreased, thus suggesting that PKM2 enters the nucleus under R848 stimulation. Similarly, the immunofluorescence more intuitively confirmed this phenomenon. All these results suggested that both dimer and tetramer of PKM2 participated in the development of NPSLE.

Microglia serve as first responders to neuronal damage and infections to restore/maintain homeostasis and have a critical role in neural circuit connectivity [101]. Previous studies have shown histological evidence of perivascular microglial activation [102, 103] in SLE patients and SLE mouse models based on positive microglial staining of CD68 and Iba-1 antigens [104]. Damaged neurons caused by synapse loss are closely associated with anomalous cognition, mainly learning and memory dysfunction, which is regulated by the hippocampus [105, 106]. In a recent study, excessive synaptic pruning by microglia was associated with the behavioral deficits of the 564Igi SLE mouse model, which displayed anxiety and cognitive defects [107]. Supposedly, microglia depletion ameliorated disease in multiple models of NPSLE [108]. Herein, we found that microglia proliferated and were activated in the hippocampus and cortex of MRL/lpr mice. Meanwhile, microglial phagocytosis was enhanced, thereby resulting in the loss of neurons and the occurrence of MRL/lpr cognitive dysfunction. We speculated that this might be related to the abnormal expression of PKM2 on these microglia. To better prove the correlation between microglial PKM2 upregulation and hippocampal neuron damage or cognitive dysfunction in lupus, AAV9-shPKM2 virus, targeting microglia, was stereotactically injected into the lateral ventricle of MRL/lpr mice to specifically downregulate PKM2 on microglia, after which morphological alterations of neurons in the hippocampus were examined, and hippocampus-dependent spatial learning and memory and contextual memory were also analyzed by MWM and fear-conditioning paradigm [109, 110]. The results showed that microglial PKM2 inhibition alleviated cognitive disorders and brain damage in MRL/lpr mice.

In addition to brain damage and cognitive improvement after microglial PKM2 inhibition, the expression of IL-6 and IL-1β in the hippocampus of MRL/lpr mice, were also reversed. It has been reported that increased serum and hippocampal levels of IL-6 and IL-1β are consistent with the progression of the lupus-like disease [111, 112]. IL-6 and IL-1β alter emotional reactivity and clinical behavior, provoking sickness and induce impairments in spatial learning experimentally [66, 67]; therefore, they could be used as evaluation indicators of neuroinflammation in the hippocampus of MRL/lpr mice. The downregulation of IL-6 and IL-1β in the hippocampus following AAV9-shPKM2 administration in MRL/lpr mice indicated that microglial PKM2 inhibition could improve neuroinflammation in MRL/lpr mice.

CNS lupus is a heterogeneous disease with many symptoms and causes. Our data suggested that the upregulation of PKM2-mediated glycolysis enhances the activation and phagocytosis of microglia, resulting in the loss of neuronal synapses and inducing cognitive dysfunction in lupus. Together, these findings suggested a novel mechanism for CNS lupus and provided a rationale for expanding future clinical trials to include CNS lupus patients, especially those with detectable glycolysis signatures.

## Conclusions

Our data suggest that PKM2 has an important role in the development of cognitive impairment in NPSLE mice. PKM2 upregulated glycolysis in the brain tissue of NPSLE mice and increased the activation of microglia and the ability of phagocytizing neuronal synapses, leading to neuronal loss and cognitive dysfunction in lupus mice. Inhibiting the expression of microglial PKM2 alleviated cognitive impairment and brain damage in NPSLE mice. These phenomena indicated that PKM2 might become a potential therapeutic target for the treatment of lupus encephalopathy.

## Supplementary Information

**Additional file 1: Supplemental figures. Fig. S1.** PCA model score scatter plot and OPLS-DA model show the separation of glycolysis metabolites in hippocampus of control mice (blue) and MLR/lpr group mice (red). All samples were included in this analysis. The horizontal ordinate (PC [1]) indicates the first principal component, and the vertical ordinate (PC [2]) indicates the second principal component. **Fig. S2.** The glycolytic activity of hippocampus and cortex of IMQ-induced mice might be related to the high expression of PKM2. (A) Glucose levels and lactate production in the hippocampus and cerebral cortex of IMQ-induced mice were normalized to that of the control group. (B) The mRNA expression of *GLUT1*, *LDHA*, and *PDK1* in the hippocampus of control and IMQ-induced groups. (C) The mRNA expression of *GLUT1*, *LDHA*, and *PDK1* in the celebrate cortex of IMQ-induced mice to the control group mice. Data represent the mean scores ± SEM. *P ≤ 0.05, **P ≤ 0.01, ***P ≤ 0.001. *n* = 6 mice per group. **Fig. S3.** The expression of PKM2 increases in IMQ-induced mice. (A) The mRNA expression of *PKM2*, *HK1*, *PFKFB3*, and *ENO1* in hippocampus of IMQ-induced mice to the control group mice. (B) The mRNA expression of *PKM2*, *HK1*, *PFKFB3*, and *ENO1* in celebrate cortex of in IMQ-induced mice to the control group mice. Data represent the mean scores ± SEM. **P ≤ 0.01. *n* = 6 mice per group. **Fig. S4.** The expression of dimeric and tetrameric forms of PKM2. (A) The abundance of the monomeric, dimeric and tetrameric forms of PKM2 are presented by the image with a short exposure (30 s). (B) The monomeric and dimeric forms are best visualized by the image with a long exposure (100 s). (C) Western blot quantification of total PKM2 and cytoplasmic PKM2 in BV2 cells treated or not treated with R848 agonist. (D) The expression levels of PKM2 (green), DAPI (blue), and their colocalization in BV2 cells treated or not treated with R848 agonist by IF staining; Bar = 20 μm. **Fig. S5.** Experimental procedure of flow cytometry analysis. (A) The schematic of sample preparation before flow cytometry detection of mouse hippocampal microglia. (B) Gating strategy of microglia (CD45^lo^CD11b^+^) in the hippocampus by flow cytometry. **Fig. S6.** Microglia is activated and PKM2 is highly expressed in hippocampal tissue of IMQ-induced mice. (A) The percentage of microglia (CD45^lo^CD11b^+^) in the hippocampus of IMQ-induced mice to the control group mice. (B) The expression level of CD86 in microglia of IMQ-induced mice to the control group mice were detected by flow cytometry. (C) The expression level of LAMP1 in the microglia of control and IMQ-induced mice t by flow cytometry. (D) The expression levels of CD68 (green), IBA-1 (red), and their colocalization (yellow) in the hippocampus of control and IMQ-induced mice were detected by IF staining, Bar = 20 μm. (E) WB quantification of IBA-1 and CD68 in hippocampus of IMQ-induced mice and control group mice. (F) The mRNA expressions of *PKM2* in the hippocampal of IMQ-induced mice and control group mice. (G) WB quantification of PKM2 in hippocampus of IMQ-induced mice and control group mice. Data represent the mean scores ± SEM. *P ≤ 0.05, **P ≤ 0.01. *n* = 6 mice per group. **Fig. S7.** TLR7 activator enhances phagocytic activity of microglia and the nuclear transfer of PKM2. (A) The expression level of LAMP1 was detected by flow cytometry with or without R848 treated for 24 h. (B) Western blot quantification of LAMP1 in BV2 cells treated with different concentrations of R848 (50, 100, 200 ng/ml) for 24 h. (C) Phagocytic function test. BV2 cells treated with different concentrations of R848 (50, 100, 200 ng/ml) for 24 h, then BV2 cells cultured with DMEM which contained fluorescent beads in incubator for 2 h. Collected the BV2 cells and detected by flow cytometry. (D) BV2 cells treated with different concentrations of R848 (50, 100 ng/ml) for 24 h. Western blot analysis of the protein expression of PSD95 after HT22 cells co-cultured with different treated BV2 cells. (E) BV2 cells treated with different concentrations of R848 (50, 100, 200 ng/ml) for 24 h, then the mRNA expressions of *PKM2* were detected by RT-qPCR. (F) BV2 cells treated with different concentrations of R848 (50, 100, 200 ng/ml) for 24 h, then the protein expressions of PKM2 were detected by western blotting. (G) BV2 cells treated with R848 (100 ng/ml) for different times (12 h, 24 h, 36 h), then the mRNA expressions of *PKM2* were detected by RT-qPCR. Data represent the mean scores ± SEM. *P ≤ 0.05, **P ≤ 0.01, ***P ≤ 0.001. *n* = 3. **Fig. S8.** TLR7 activates the PKM2/b-catenin pathway to enhance the phagocytic activity of microglia. (A) The mRNA expression level of *c-Myc*, *Axin-2* and *Cyclin-D1* were detected by RT-qPCR in BV2 cells treated with 100 ng/ml R848 for different time (12 h, 24 h, 72 h), and analyzed the correlation between *PKM2* and *c-Myc*, *Axin-2* and *Cyclin-D1*. (B) The mRNA expression level of *c-Myc*, *Axin-2* and *Cyclin-D1* were detected by RT-qPCR in BV2 cells treated with different concentrations of R848 (50, 100, 200 ng/ml) for 24 h, and analyzed the correlation between *PKM2* and *c-Myc*, *Axin-2* and *Cyclin-D1*. (C) Western blot quantification of β-Catenin, c-Myc and Cyclin-D1 in BV2 cells treated with different concentrations of R848 (50, 100, 200 ng/ml) for 24 h. (D) The expression levels of β-Catenin (Green) in BV2 cells treated with 100 ng/ml R848 by if staining, Bar = 20 μm. (E) Phagocytic function test. BV2 cells induced with 100 ng/ml R848 for 24 h, then treated with or without 200 ng/ml β-Catenin inhibitor (KYA1797K) for 24 h. All group BV2 cells were cultured with DMEM which contained fluorescent beads in incubator for 2 h. Collected the BV2 cells and detected by flow cytometry. (F) BV2 cells induced with 100 ng/ml R848 for 24 h, then treated with or without 200 ng/ml β-Catenin inhibitor (KYA1797K) for 24 h. The expression level of LAMP1 in BV2 cells was detected by flow cytometry. (G) BV2 cells induced with 100 ng/ml R848 for 24 h, then treated with or without 200 ng/ml β-Catenin inhibitor (KYA1797K) for 24 h. Western blot analysis of the protein expression of PSD95 after HT22 cells co-cultured with different treated BV2 cells. Data represent the mean scores ± SEM. *P ≤ 0.05, **P ≤ 0.01, ***P ≤ 0.001. *n* = 3. **Figure S9.** Verification of PKM2 conditional knockout. (A) Schematic diagram of virus structure. (B) The expression levels of PKM2 (red), GFAP (white), and their colocalization in the hippocampus detected by IF staining, Bar = 20 μm. (C) The expression levels of PKM2 (red), NeuN (white), and their colocalization in the hippocampus detected by IF staining, Bar = 20 μm. (D) The expression levels of PKM2 (red), IBA-1 (white), and their colocalization in the hippocampus of vector group and shPKM2 group mice detected by IF staining, Bar = 20 μm. **Fig. S10.** Route of mouse water maze. (A) Morris water maze test. Representative swimming traces of mice from different groups on the first day and the fifth training day. The hidden platform is located in quadrant III. (B) The time latency to find the hidden platform in different groups of mice during consecutive 5 training days. (C) The swimming trajectory of the test mice on day 6 of the water maze experiment.

## Data Availability

The datasets used and/or analyzed during the current study are available from the corresponding author on reasonable request.

## Abbreviations

NPSLE: Neuropsychiatric systemic lupus erythematosus
PKM2: Pyruvate kinase isoform M2
SLE: Systemic lupus erythematosus
DNRAb: Anti-DNA antibodies
CNS: Central nervous system
BSA: Bovine serum albumin
DAPI: 2-(4-Amidinophenyl)-1H-indole-6-carboxamidine
SDS-PAGE: Sodium dodecyl sulfate-polyacrylamide gel electrophoresis
IMQ: Imiquimod
ELISA: Enzyme-linked immunosorbent assay
MWM: Morris water maze
PGI: Phosphoglucose isomerase
PFK: Phosphofructokinase-1
DHAP: Dihydroxyacetone phosphate
TPI: Triosephosphate isomerase
HK1: Hexokinase 1
PFKFB3: 6-Phosphofructo-2-kinase/fructose-2,6-bisphosphatase 3
ENO1: Enolase 1
LDHA: Lactate dehydrogenase A
GLUT1: Glucose transporter 1
LDHA: Lactic dehydrogenase kinase A
PDK1: Pyruvate dehydrogenase kinase 1
LAMP1: Lysosome-associated membrane protein 1
VGLUT1: Vesicular glutamate transporter 1
CA: Cornu ammonis
DG: Dentate gyrus
BBB: Brain blood–brain barrier
CSF: Cerebrospinal fluid
MFI: Mean fluorescence intensity
