# Supplementary material for: Pyruvate kinase isoform M2 impairs cognition in systemic lupus erythematosus by promoting microglial synaptic pruning via the β-catenin signaling pathway
Source: J Neuroinflammation. 2021 Oct 13;18:229. doi: 10.1186/s12974-021-02279-9 (PMC8513209; doi:10.1186/s12974-021-02279-9)
Supplement: Supplementary file 1 — Additional file 1: Supplemental figures. Fig. S1. PCA model score scatter plot and OPLS-DA model show the separation of glycolysis metabolites in hippocampus of control mice (blue) and MLR/lpr group mice (red). All samples were included in this analysis. The horizontal ordinate (PC [1]) indicates the first principal component, and the vertical ordinate (PC [2]) indicates the second principal component. Fig. S2. The glycolytic activity of hippocampus and cortex of IMQ-induced mice might be related to the high expression of PKM2. (A) Glucose levels and lactate production in the hippocampus and cerebral cortex of IMQ-induced mice were normalized to that of the control group. (B) The mRNA expression of GLUT1, LDHA, and PDK1 in the hippocampus of control and IMQ-induced groups. (C) The mRNA expression of GLUT1, LDHA, and PDK1 in the celebrate cortex of IMQ-induced mice to the control group mice. Data represent the mean scores ± SEM. *P ≤ 0.05, **P ≤ 0.01, ***P ≤ 0.001. n = 6 mice per group. Fig. S3. The expression of PKM2 increases in IMQ-induced mice. (A) The mRNA expression of PKM2, HK1, PFKFB3, and ENO1 in hippocampus of IMQ-induced mice to the control group mice. (B) The mRNA expression of PKM2, HK1, PFKFB3, and ENO1 in celebrate cortex of in IMQ-induced mice to the control group mice. Data represent the mean scores ± SEM. **P ≤ 0.01. n = 6 mice per group. Fig. S4. The expression of dimeric and tetrameric forms of PKM2. (A) The abundance of the monomeric, dimeric and tetrameric forms of PKM2 are presented by the image with a short exposure (30 s). (B) The monomeric and dimeric forms are best visualized by the image with a long exposure (100 s). (C) Western blot quantification of total PKM2 and cytoplasmic PKM2 in BV2 cells treated or not treated with R848 agonist. (D) The expression levels of PKM2 (green), DAPI (blue), and their colocalization in BV2 cells treated or not treated with R848 agonist by IF staining; Bar = 20 μm. Fig. S5. Experimental procedure of flow cy [file 12974_2021_2279_MOESM1_ESM.docx]

**Pyruvate kinase isoform M2 impairs cognition in systemic lupus erythematosus by promoting microglial synaptic pruning via β-catenin signaling pathway**

**Li Lu^1,2#^, Hailin Wang^1,2#^, Xuan Liu^1,2^, Liping Tan^1,2^, XiaoYue Qiao^1,2^, Jiali Ni^1,2^, Yang Sun^4*^, Jun Liang^3*^, Yayi Hou^1,2,3*^, Huan Dou^1,2,3*^**

1. The State Key Laboratory of Pharmaceutical Biotechnology, Division of Immunology, Medical School, Nanjing University, Nanjing 210093, PR China

2. Jiangsu Key Laboratory of Molecular Medicine, Nanjing, 210093, PR China, 210093

3. Department of Rheumatology and Immunology, Nanjing Drum Tower Hospital, The Affiliated Hospital of Nanjing University Medical School, Nanjing 210008, PR China.

4. The State Key Laboratory of Pharmaceutical Biotechnology and Collaborative Innovation Center of Chemistry for Life Sciences, School of Life Sciences, Nanjing University, Nanjing, 210023, China.

# Li Lu and Hailin Wang contributed equally to this article.

*Corresponding author:

Huan Dou, Email: [douhuan@nju.edu.cn](mailto:douhuan@nju.edu.cn); Yayi Hou, Email: yayihou@nju.edu.cn; Jun Liang, Email: [13505193169@139.com](mailto:13505193169@139.com); Yang Sun, Email: yangsun@nju.edu.cn.

**1. Materials and methods**

**1.1 Animals**

Female C57BL/6 mice were obtained from the Model Animal Research Center of Nanjing University (Nanjing, China) and were kept in specific pathogen-free (SPF) conditions. The Nanjing University Animal Care Commission approved all manipulations. For lupus model induction, 7- to 8-week-old female C57BL/6 mice were topically treated with 5% IMQ cream (3M Health Care, St. Paul, MN, USA) for 2 months as described previously. In brief, 7-9-week-old female C57BL/6 mice were treated topically with Imiquimod Cream (Keyi, Hubei, China) to the ears three times weekly. The mice were killed after 10 weeks IMQ treatment. All the mice were housed in specific pathogen-free conditions at the Nanjing University Animal Care Commission.


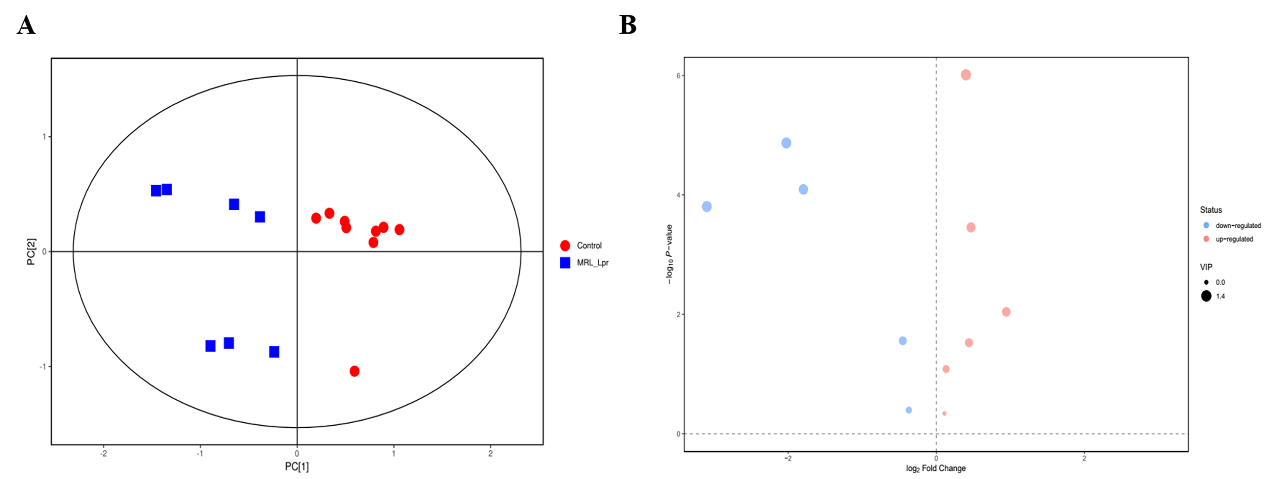


**Figure S1. PCA model score scatter plot and OPLS-DA model show the separation of glycolysis metabolites in hippocampus of control mice (blue) and MLR/lpr group mice (red).** All samples were included in this analysis. The horizontal ordinate (PC [1]) indicates the first principal component, and the vertical ordinate (PC [2]) indicates the second principal component.


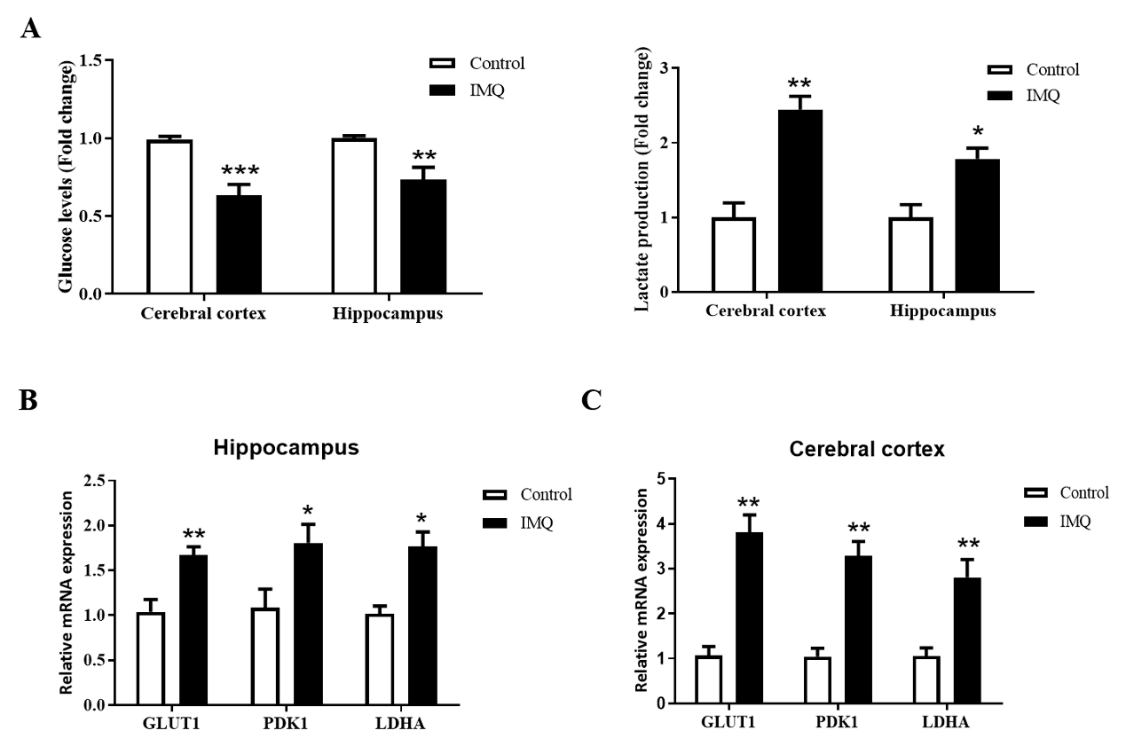


**Figure S2. The glycolytic activity of hippocampus and cortex of IMQ-induced mice might be related to the high expression of PKM2. (A)** Glucose levels and lactate production in the hippocampus and cerebral cortex of IMQ-induced mice were normalized to that of the control group. **(B)** The mRNA expression of *GLUT1*, *LDHA*, and *PDK1* in the hippocampus of control and IMQ-induced groups. **(C)** The mRNA expression of *GLUT1*, *LDHA*, and *PDK1* in the celebrate cortex of IMQ-induced mice to the control group mice. Data represent the mean scores ± SEM. *P ≤ 0.05, **P ≤ 0.01, ***P ≤ 0.001. n = 6 mice per group.


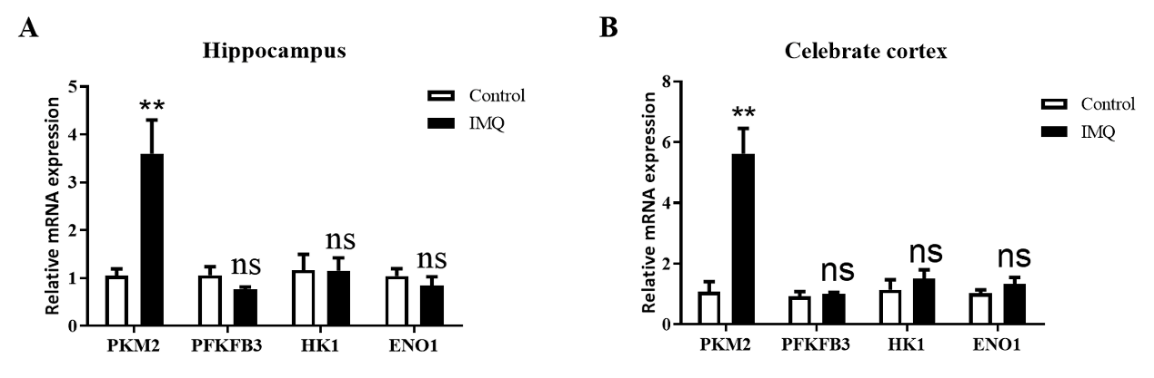


**Figure S3. The expression of PKM2 increases in IMQ-induced mice. (A)** The mRNA expression of *PKM2*, *HK1*, *PFKFB3*, and *ENO1* in hippocampus of IMQ-induced mice to the control group mice. **(B)** The mRNA expression of *PKM2*, *HK1*, *PFKFB3*, and *ENO1* in celebrate cortex of in IMQ-induced mice to the control group mice. Data represent the mean scores ± SEM. **P ≤ 0.01. n = 6 mice per group.


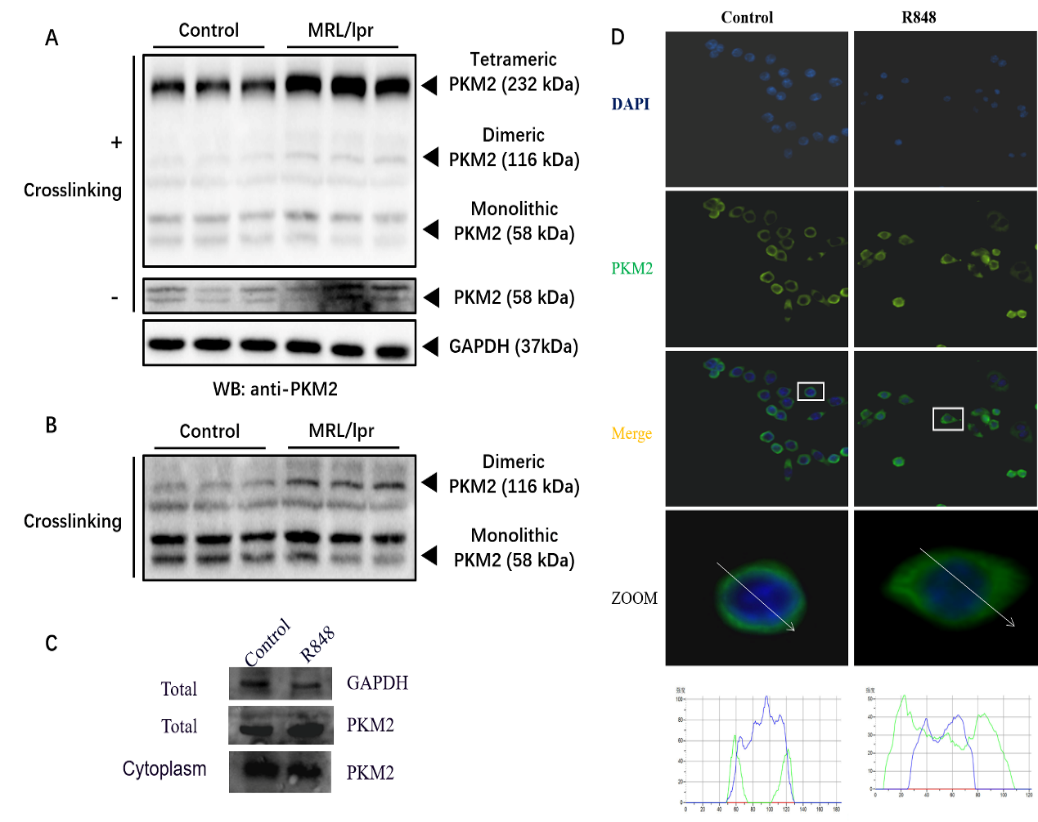


**Figure S4. The expression of dimeric and tetrameric forms of PKM2. (A)** The abundance of the monomeric, dimeric and tetrameric forms of PKM2 are presented by the image with a short exposure (30 s). **(B)** The monomeric and dimeric forms are best visualized by the image with a long exposure (100 s). **(C)** Western blot quantification of total PKM2 and cytoplasmic PKM2 in BV2 cells treated or not treated with R848 agonist. **(D)** The expression levels of PKM2 (green), DAPI (blue), and their colocalization in BV2 cells treated or not treated with R848 agonist by IF staining; Bar = 20 μm.


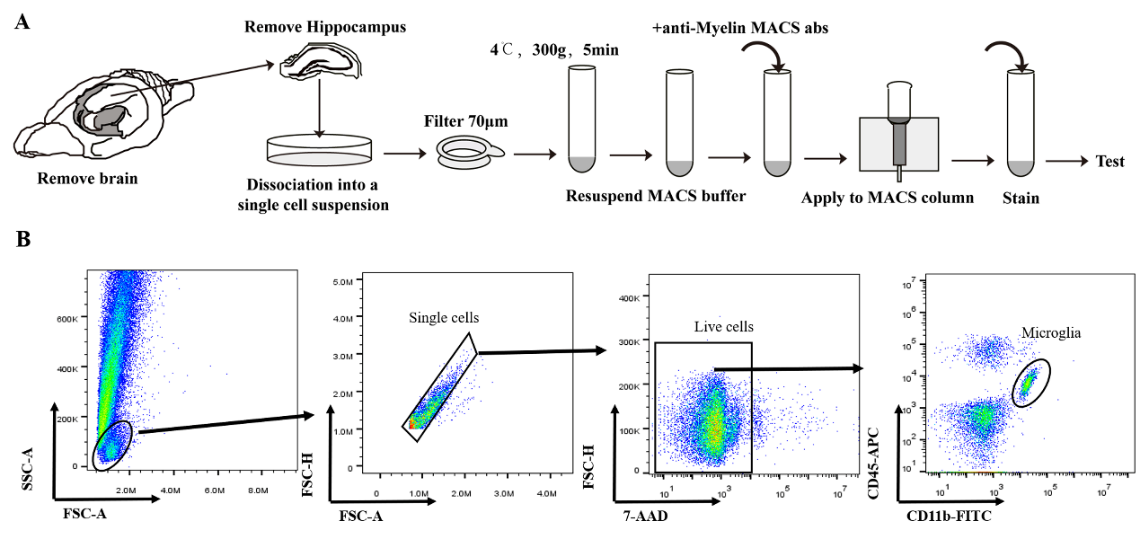


**Figure S5. Experimental procedure of flow cytometry analysis. (A)** The schematic of sample preparation before flow cytometry detection of mouse hippocampal microglia. **(B)** Gating strategy of microglia (CD45^lo^CD11b^+^) in the hippocampus by flow cytometry.


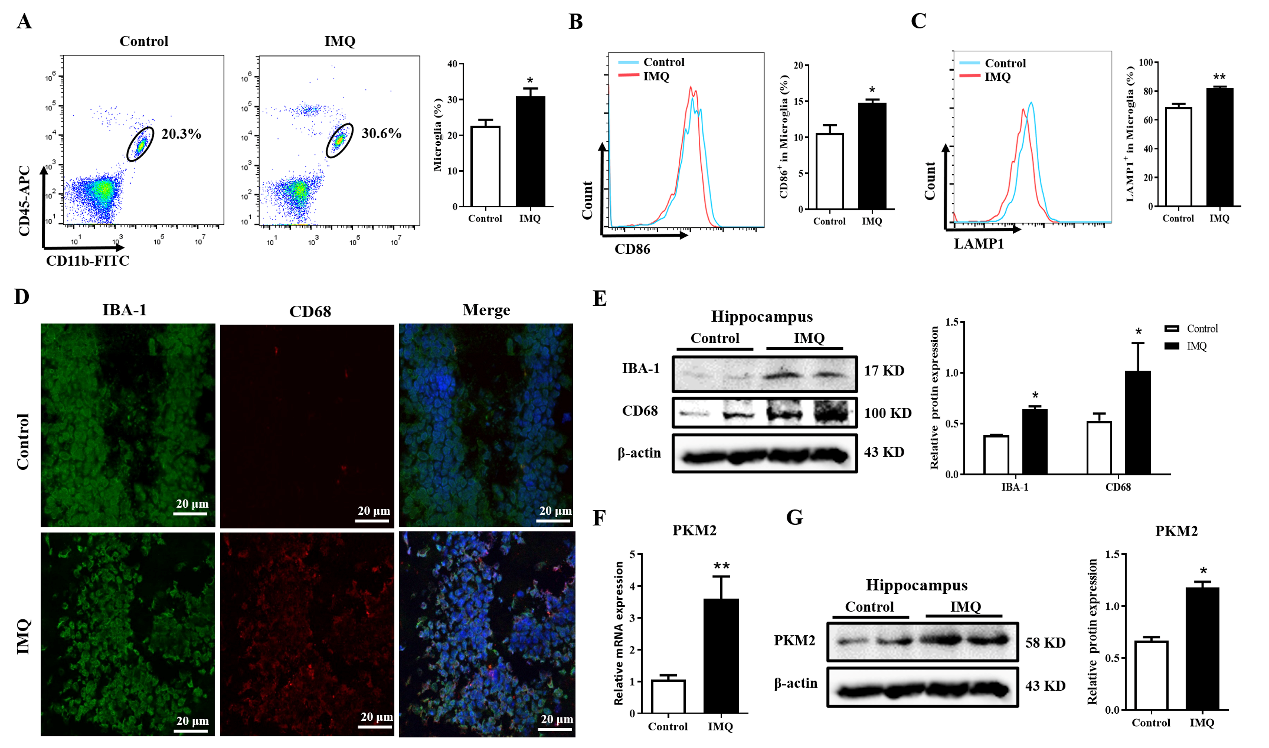


**Figure S6 Microglia is activated and PKM2 is highly expressed in hippocampal tissue of IMQ-induced mice. (A)** The percentage of microglia (CD45^lo^CD11b^+^) in the hippocampus of IMQ-induced mice to the control group mice. **(B)** The expression level of CD86 in microglia of IMQ-induced mice to the control group mice were detected by flow cytometry. **(C)** The expression level of LAMP1 in the microglia of control and IMQ-induced mice t by flow cytometry. **(D)** The expression levels of CD68 (green), IBA-1 (red), and their colocalization (yellow) in the hippocampus of control and IMQ-induced mice were detected by IF staining, Bar = 20μm. **(E)** WB quantification of IBA-1 and CD68 in hippocampus of IMQ-induced mice and control group mice. **(F)** The mRNA expressions of *PKM2* in the hippocampal of IMQ-induced mice and control group mice. **(G)** WB quantification of PKM2 in hippocampus of IMQ-induced mice and control group mice. Data represent the mean scores ± SEM. *P ≤ 0.05, **P ≤ 0.01. n = 6 mice per group.


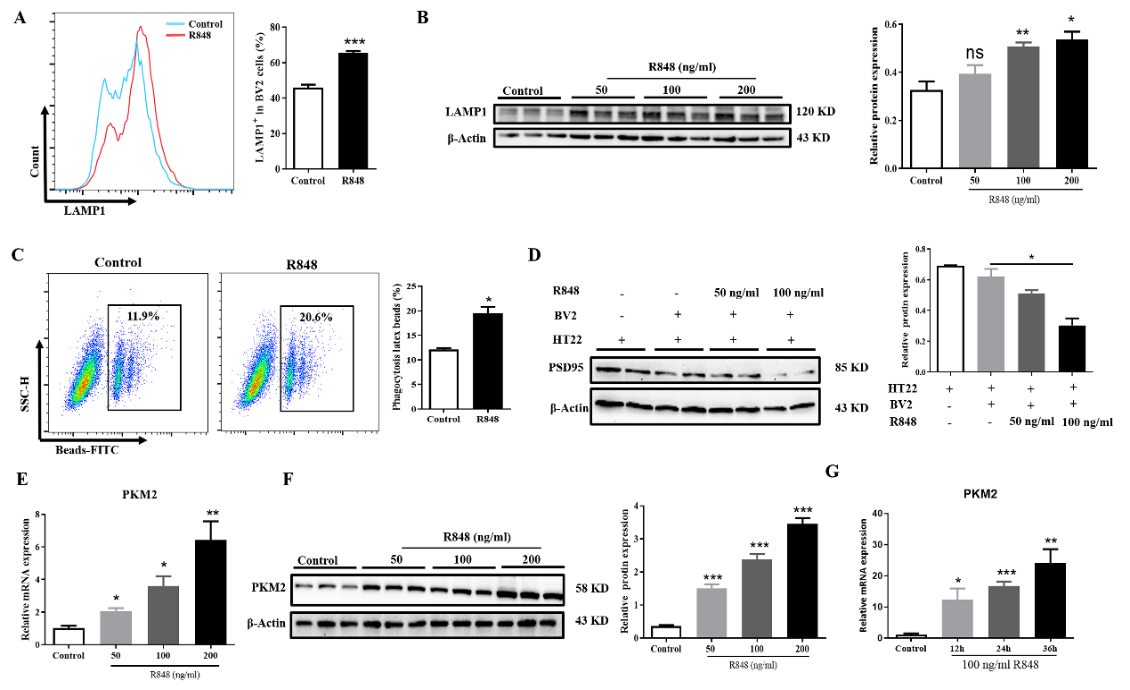


**Figure S7 TLR7 activator enhances phagocytic activity of microglia and the nuclear transfer of PKM2.** **(A)** The expression level of LAMP1 was detected by flow cytometry with or without R848 treated for 24h. **(B)** Western blot quantification of LAMP1 in BV2 cells treated with different concentrations of R848 (50, 100, 200 ng/ml) for 24h. **(C)** Phagocytic function test. BV2 cells treated with different concentrations of R848 (50, 100, 200 ng/ml) for 24h, then BV2 cells cultured with DMEM which contained fluorescent beads in incubator for 2 h. Collected the BV2 cells and detected by flow cytometry. **(D)** BV2 cells treated with different concentrations of R848 (50, 100 ng/ml) for 24h. Western blot analysis of the protein expression of PSD95 after HT22 cells cocultured with different treated BV2 cells. **(E)** BV2 cells treated with different concentrations of R848 (50, 100, 200 ng/ml) for 24h, then the mRNA expressions of *PKM2* were detected by RT-qPCR. **(F)** BV2 cells treated with different concentrations of R848 (50, 100, 200 ng/ml) for 24h, then the protein expressions of PKM2 were detected by western blotting. **(G)** BV2 cells treated with R848 (100 ng/ml) for different times (12h, 24h, 36h), then the mRNA expressions of *PKM2* were detected by RT-qPCR. Data represent the mean scores ± SEM. *P ≤ 0.05, **P ≤ 0.01, ***P ≤ 0.001. n = 3.


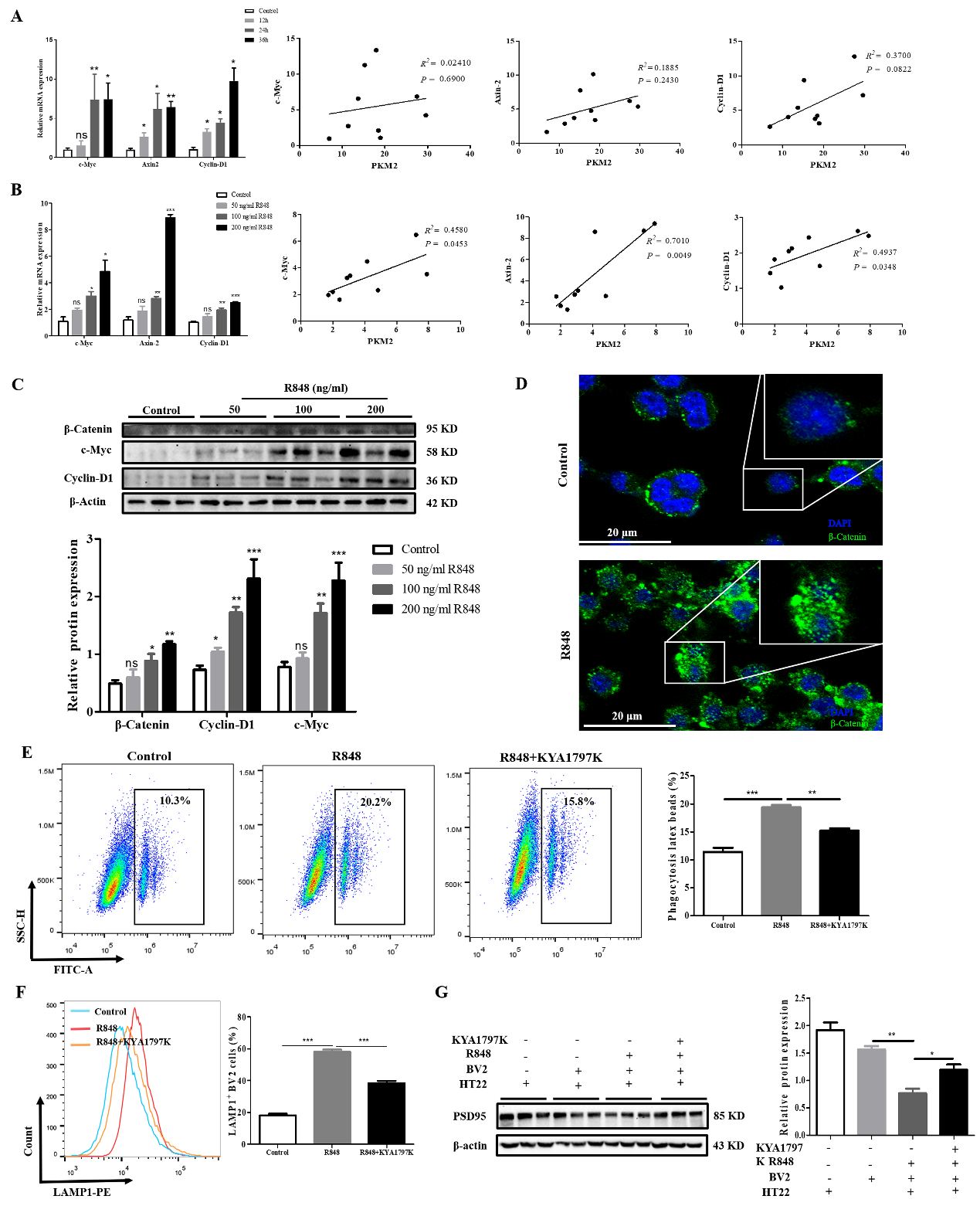


**Figure S8. TLR7 activates the PKM2/b-catenin pathway to enhance the phagocytic activity of microglia.** **(A)** The mRNA expression level of *c-Myc*, *Axin-2* and *Cyclin-D1* were detected by RT-qPCR in BV2 cells treated with 100ng/ml R848 for different time (12h, 24h, 72h), and analyzed the correlation between *PKM2* and *c-Myc*, *Axin-2* and *Cyclin-D1*. **(B)** The mRNA expression level of *c-Myc*, *Axin-2* and *Cyclin-D1* were detected by RT-qPCR in BV2 cells treated with different concentrations of R848 (50, 100, 200 ng/ml) for 24h, and analyzed the correlation between *PKM2* and *c-Myc*, *Axin-2* and *Cyclin-D1*. **(C)** Western blot quantification of β-Catenin, c-Myc and Cyclin-D1 in BV2 cells treated with different concentrations of R848 (50, 100, 200 ng/ml) for 24h. **(D)** The expression levels of β-Catenin (Green) in BV2 cells treated with 100ng/ml R848 by if staining, Bar = 20μm. **(E)** Phagocytic function test. BV2 cells induced with 100ng/ml R848 for 24h, then treated with or without 200 ng/ml β-Catenin inhibitor (KYA1797K) for 24 hours. All group BV2 cells were cultured with DMEM which contained fluorescent beads in incubator for 2 h. Collected the BV2 cells and detected by flow cytometry. **(F)** BV2 cells induced with 100ng/ml R848 for 24h, then treated with or without 200 ng/ml β-Catenin inhibitor (KYA1797K) for 24 hours. The expression level of LAMP1 in BV2 cells was detected by flow cytometry. **(G)** BV2 cells induced with 100ng/ml R848 for 24h, then treated with or without 200 ng/ml β-Catenin inhibitor (KYA1797K) for 24 hours. Western blot analysis of the protein expression of PSD95 after HT22 cells cocultured with different treated BV2 cells. Data represent the mean scores ± SEM. *P ≤ 0.05, **P ≤ 0.01, ***P ≤ 0.001. n = 3.


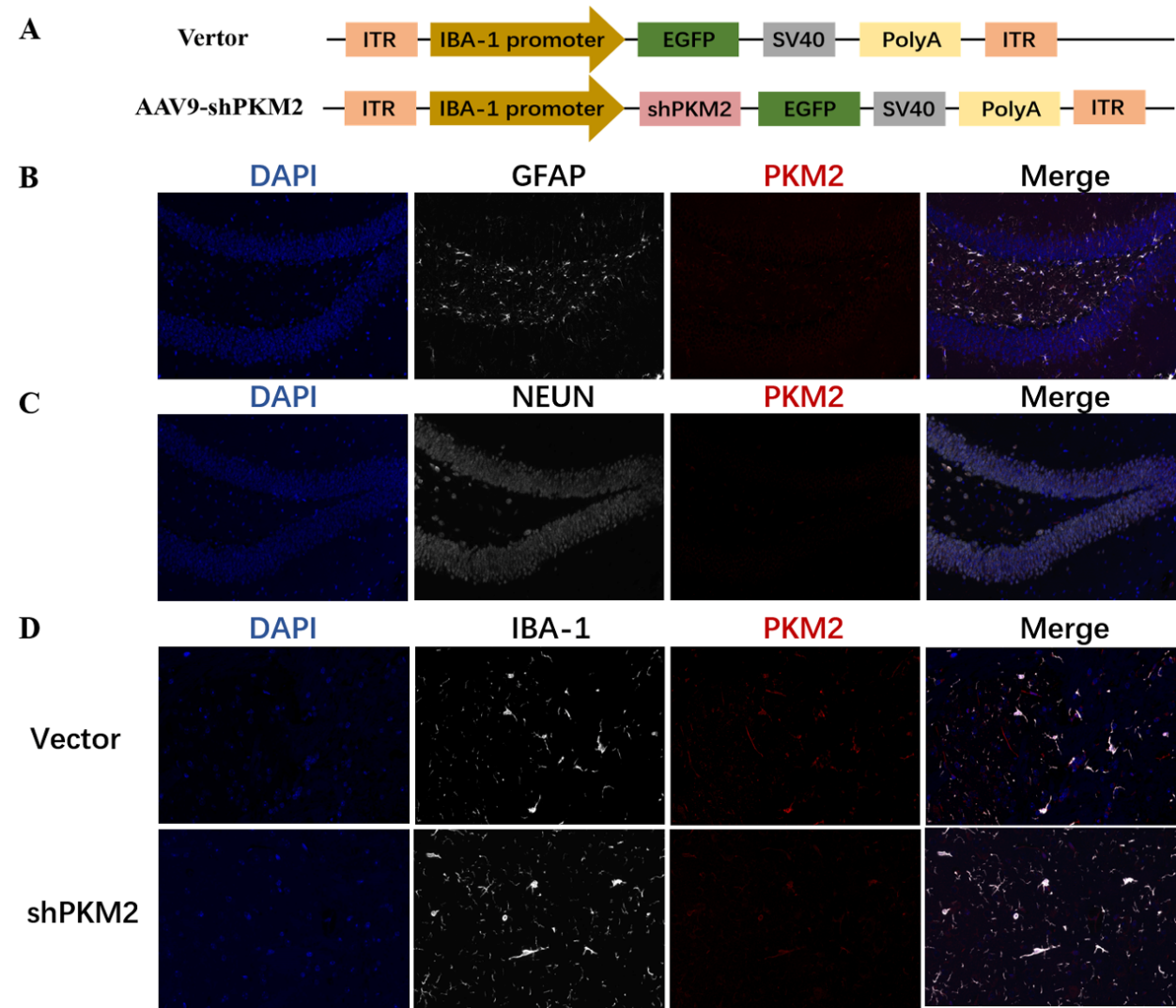


**Figure S9: Verification of PKM2 conditional knockout. (A)** Schematic diagram of virus structure. **(B)** The expression levels of PKM2 (red), GFAP (white), and their colocalization in the hippocampus detected by IF staining, Bar = 20μm. **(C)** The expression levels of PKM2 (red), NeuN (white), and their colocalization in the hippocampus detected by IF staining, Bar = 20μm. **(D)** The expression levels of PKM2 (red), IBA-1 (white), and their colocalization in the hippocampus of vector group and shPKM2 group mice detected by IF staining, Bar = 20μm.


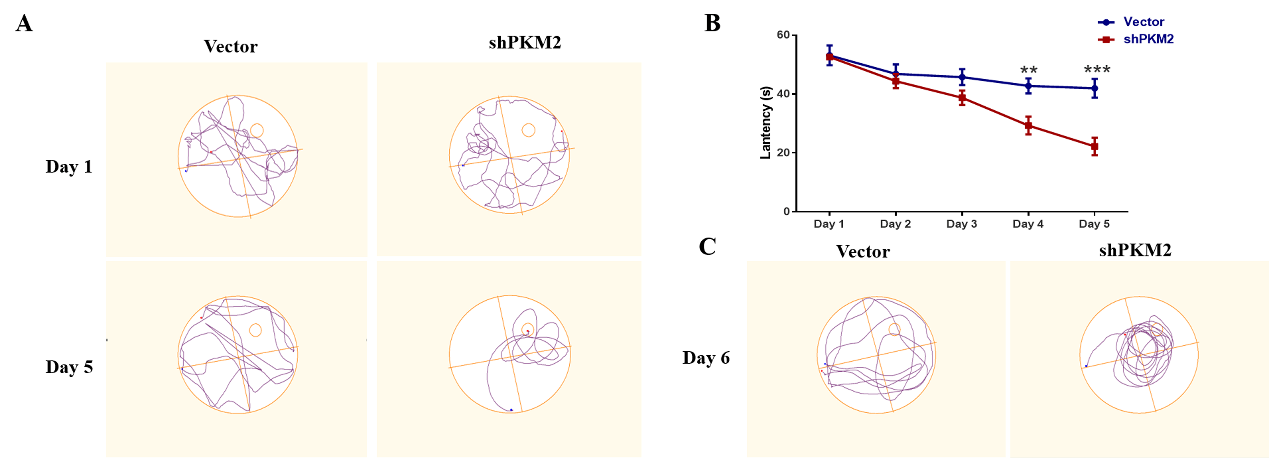


**Figure S10:** **Route of mouse water maze. (A)** Morris water maze test. Representative swimming traces of mice from different groups on the first day and the fifth training day. The hidden platform is located in quadrant III. **(B)** The time latency to find the hidden platform in different groups of mice during consecutive 5 training days. **(C)** The swimming trajectory of the test mice on day 6 of the water maze experiment.
